# Surgery-induced gut microbial dysbiosis promotes cognitive impairment via regulation of intestinal function and the metabolite palmitic amide

**DOI:** 10.1186/s40168-023-01689-6

**Published:** 2023-11-08

**Authors:** Cailong Pan, Huiwen Zhang, Lingyuan Zhang, Lu Chen, Lu Xu, Ning Xu, Xue Liu, Qinghai Meng, Xiaoliang Wang, Zhi-Yuan Zhang

**Affiliations:** 1 School of Basic Medical Sciences, Nanjing Medical University, Longmian Avenue 101, Nanjing, 211166 China; 2 School of Medicine & Holistic Integrative Medicine, Nanjing University of Chinese Medicine, Nanjing, 210023 China; 3 Department of Anesthesiology, Nanjing First Hospital, Nanjing Medical University, Changle Road 89, Nanjing, 210029 China; 4 Key Laboratory of Rare Metabolic Diseases, Nanjing Medical University, Nanjing, 211166 China

**Keywords:** Perioperative neurocognitive disorders, Gut microbiota, Microbiota, Fecal microbiota transplantation, Intestinal permeability, Palmitic amide

## Abstract

**Background:**

Perioperative neurocognitive disorders (PND) are the most common postoperative complications with few therapeutic options. Gut microbial dysbiosis is associated with neurological diseases; however, the mechanisms by which the microbiota regulates postoperative gastrointestinal and cognitive function are incompletely understood.

**Methods:**

Behavioral testing, MiSeq 16S rRNA gene sequencing, non-target metabolism, intestinal permeability detection, protein assays, and immunofluorescence staining were employed to discern the impacts of surgery on microbial profiles, intestinal barriers, serum metabolism, and the brain. Interventions in mice included fecal microbiota transplantation, the anti-inflammatory agent dexamethasone, *Lactobacillus* supplementation, indole propionic acid supplementation, and palmitic amide administration.

**Results:**

Surgery-induced cognitive impairment occurs predominantly in aged mice, and surgery-induced alterations in the microbiota composition profile exacerbate intestinal barrier disruption in aged mice. These adverse effects can be mitigated by transferring microbiota from young donors or by bolstering the intestinal barrier function using dexamethasone, *Lactobacillus*, or indole propionic acid. Moreover, microbiota composition profiles can be restored by transplanting feces from young mice to aged surgical mice, improving neuropathology and cognitive function, and these effects coincide with increased intestinal permeability. Metabolomic screening identified alterations in metabolites in mouse serum after surgery, especially the increase in palmitic amide. Palmitic amide levels in serum and brain can be decreased by transplanting feces from young mice to aged surgical mice. Oral palmitic amide exacerbates cognitive impairment and neuropathological changes in mice.

**Conclusions:**

Gut microbial dysbiosis in mice after surgery is a key mechanism leading to cognition dysfunction, which disrupts the intestinal barrier and metabolic abnormalities, resulting in neuroinflammation and dendritic spine loss. Intestinal barrier damage and high level of palmitic amide in old mice may be the cause of high incidence of PND in the elderly. Preoperative microbiota regulation and intestinal barrier restoration may be of therapeutic benefit in preventing PND.

Video Abstract

**Supplementary Information:**

The online version contains supplementary material available at 10.1186/s40168-023-01689-6.

## Background

Perioperative neurocognitive disorders (PND) are the most common postoperative complications and include persistent deficits in learning function, memory, and other cognitive domains [1, 2]. Aging has been recognized as a risk factor for neurological complications after surgery, especially cognitive impairment [3]. Delirium and postoperative neurocognitive impairment have become the most common perioperative complications in patients over 65 years of age [4]. However, the pathophysiology of PND still remains unknown; therefore, exploring its pathogenesis and providing a treatment strategy could reduce the high personal and societal costs of PND.

Growing evidence shows a link between the gut microbiome and neurological diseases, with neuroinflammation and behavior modulated through fecal microbiota transplantation (FMT) [5, 6]. In rodent models of neurological diseases, such as Parkinson’s disease [7] and Alzheimer’s disease [8], disturbances in the gut microbiota lead to neuroinflammation and also promote the activation of microglia and pathological changes in brain tissue. Neuroinflammation and neuronal cell damage in brain tissues are the most critical features of PND [9]. A recent review discussed that the gut microbiota may contribute to the development of postoperative cognitive dysfunction through the gut-brain axis [10], which is a bidirectional communication system between the gut and brain [11]. Multiple perioperative stimuli (including anesthetics, postoperative trauma and pain) could cause short- or long-term effects on the gut microbiota, including compositional changes in diversity and delays in colonization time [10]. In addition to surgical factors, aging is also an independent factor, which may be due to profound differences between aging and young individuals, such as decreased reserve and function of multiple physiological systems, including intestinal permeability and microbial dysregulation [12, 13], but the degree to which these differences promote or prevent PND is unclear. Therefore, exploring the mechanism of gut microbiota in PND could provide a unique point of perioperative interventions in elderly.

To investigate the impact of surgery-associated microbiota composition on the gut and brain at different age states, we used young (2 months) and aged (18 months) mice. We compared the effects of surgery on intestinal permeability in young and aged mice and performed fecal microbiota transplantation (FMT) to explore whether FMT could prevent the increase of intestinal permeability in aged mice that underwent surgery, and observed the effect of FMT on symptoms of PND. Our recent research has demonstrated that indole propionic acid (IPA) augments the intestinal barrier function in diabetic mice [14], and it is regarded as a beneficial metabolite derived from gut microbiota. Furthermore, we administered indole propionic acid, *Lactobacillus*—widely acknowledged as a probiotic—and dexamethasone, all believed to possess anti-inflammatory properties, as preventive measures in aged mice. We subsequently observed their impacts on the gut microbiota, intestinal permeability, and cognitive function in elderly postoperative mice. To determine how surgery-induced changes in gut microbiota contribute to cognitive dysfunction in mice, we screened numerous aberrant metabolites from the metabolite profiles of mice, and identified the metabolite palmitic amide (PA) as a key compound responsible for PND.

## Methods

### Animal management

Adult male C57BL/6 mice (2 and 18 months old) were provided by the Experimental Animal Center of Nanjing Medical University, Nanjing, China. All experimental protocols and procedures were approved by the Nanjing Medical University Animal Care and Use Committee, in accordance with the National Institutes of Health Guide for the Care and Use of Laboratory Animals.

C57BL/6 mice (2 months old and 18 months old) were grouped as follows: young control, young surgery, aged control, aged surgery, aged surgery + IPA, aged surgery + DEX, aged surgery + *Lac.*, young + PA, young surgery + PA, young surgery + youth FMT, and young surgery + aged FMT groups, aged surgery + youth FMT, and aged surgery + aged FMT groups. These experiments on the different groups of mice were not conducted simultaneously. After establishing suitable control groups, these animals underwent at least 6 independent experiments.

Except for the young and aged groups, mice in all other groups underwent laparotomy. Briefly, these mice which had an operation were anesthetized with pentobarbitone (30 mg/kg), had a 2-cm wound made in the abdominal cavity, and the abdominal viscera was exposed for 5 min. Then, the peritoneal muscle and skin layers were sutured. The procedures, including anesthesia and surgery, were used as the overall conditions to induce PND; therefore, the mice in the young and aged groups did not receive any treatment.

For IPA administration, mice were supplemented daily with 100 mg/kg body weight IPA (Sigma-Aldrich, St. Louis, MO, USA) in CMC-Na (vehicle, 0.1%) orally for 4 weeks before surgery. For *Lactobacillus acidophilus* (*Lac.*) administration, mice were supplemented daily with 0.1 ml/10 g body weight *Lac.* (10^9^ CFU/ml) orally for 4 weeks before surgery. For dexamethasone (DEX) (ST1254, beyotime, China) administration, mice were supplemented daily with 1 mg/kg body weigh DEX in CMC-Na (vehicle, 0.1%) orally for 4 weeks before surgery. For PA administration, mice were supplemented daily with 50 mg/kg PA (Shanghai Yuanye Biotechnology Co., Ltd, Shanghai, China) in CMC-Na (vehicle, 0.1%) orally. The FMT groups were aged surgical mice that received fecal microbiota transplantation from young or aged mice.

Mice that received FMT receive antibiotic-laced drinking water (vancomycin 0.5 g/L + ampicillin 1 g/L) to suppress their own gut microbes for 7 days before surgery, and received FMT once a day for seven consecutive days after surgery. Specifically, the feces of the mice in the young and old groups (naïve mice) were collected at 7:00 am every day, mixed with the mice feces in the same group into a pre-deoxygenated sterile centrifuge tube, and added to sterile saline-PBS at a ratio of 100 mg:1 mL to fully suspend the feces. They were then left at room temperature until the supernatant appeared (approximately 10 min), and the supernatant was administered to recipient mice (0.1 mL/10 g) by gavage. All mice underwent behavioral tests after 7 days, including the MWM, FCT, and NORT. On the 20th postoperative day, the mice were sacrificed after collection of feces, blood, colon, and intact brain tissue was obtained. In some groups, intestinal blood flow was measured with a Doppler instrument before the mice sacrificed.

### Behavioral tests

Behavioral tests, including the Morris water maze (MWM) test, fear conditioning test (FCT), and new object recognition test (NORT), were performed as previous reports [15, 16].

#### MWM test

All the mice subjected to the MWM test underwent the first training test on the 8th day of laparotomy. Each mouse was placed into the pool in either the upper right (first quadrant), lower right (second quadrant), lower left (third quadrant), or upper left (fourth quadrant) quadrant. The time(s) for the mouse to find the underwater platform, which was in the upper left (fourth quadrant) of the pool, was recorded as latency. The swimming trajectory of the mice and other information, such as swimming distance and time, were also recorded by the camera system and Xeye animal behavior trajectory analysis system (ANY-maze, Stoelting, USA). If the time exceeded 60 s, the mice were guided to the platform and allowed to remain there for 10 s. The same training was performed on days 9–12 post-surgery in mice. On the 13th day after the operation, the underwater platform in the fourth quadrant was removed to detect spatial memory, and the mice in each group were placed in the second quadrant for 60 s of exploration. The time spent by mice in the fourth quadrant and the number of times they entered the area where the platform was located were recorded. The camera system and Xeye animal behavior trajectory analysis system was used to record the swimming trajectory and swimming distance of the mice and other information.

#### FCT

All mice were subjected to the FCT 14 days after the laparotomy using a dedicated chamber (Panlab, Harvard Apparatus, Holliston, MA, USA). Fear conditioning was established as follows: on the 14th postoperative day, the mice were placed in the chamber for 10 min without any stimulation to acclimate to the environment. On the 15th day, alternating stimulation containing sound signals (4.5 kHz, 60 dB, 30 s) and foot shock (1 mA, 5 s) was administered to the mice. On the 16th day, the mice were placed in the chamber for 5 min without any shock to the foot. SuperFC-conditioned fear video analysis software (PACKWIN 2.0.06, Harvard Apparatus, Holliston, MA, USA) automatically calculated the freezing time of the mice and calculated the freezing time as a percentage of the total time (freezing total %).

#### NORT

All mice subjected to the NOR test started on day 17 after the laparotomy were placed in a box-shaped space of 25 × 25 × 50 cm and acclimated for 10 min. On day 18, a log-colored square wooden block (object A) and a square wooden block (object B) were placed on the two ends of the same sidewall of the box-shaped space, and the mice were placed in the box with their backs against the two objects to conduct the training exploration of objects A and B. The video recorder at the top of the box was turned on to record mouse behavior for 10 min. On the 19th day, object B in the box-shaped space was replaced with object C (a red triangular wooden block), which was then placed in the box-shaped space, and the video recorder at the top of the box was turned on to record the behavior of the mice for 10 min. After reading the video, the time spent exploring the novel object was recorded. The recognition index (RI) was calculated as RI = time spent exploring the novel object/total length of time spent exploring both objects × 100%.

### Immunofluorescence and immunohistochemistry staining

Brain, ileum, and colon tissues were perfused intracardially with 4% paraformaldehyde in ice-cold PBS. The brain and colon tissues were removed, fixed again, and dehydrated in a 30% sucrose solution. The brains were processed, sectioned, and stained by immunofluorescence with the following antibodies: IBA1 (ab178847, Abcam, USA) (1:200), GFAP (3670 s, Cell Signaling Technology, USA) (1:200), and NeuN (ab177487, Abcam, USA) (1:200). The ileums were processed, sectioned, and stained by immunohistochemistry with the following antibodies: IL-17a (ab79056, Abcam, USA) (1:200) and IL-22 (ab203211, Abcam, USA) (1:200).The ileums and colons were processed, sectioned, and stained by immunofluorescence with the following antibodies: ZO-1 (ab221547, Abcam, USA) (1:200), Cluadin-1 (ab211737, Abcam, USA) (1:200), and Occludin (ab216327, Abcam, USA) (1:200). All stained sections were randomly assigned and independently analyzed by observers without knowledge of the treatments. Image-Pro Plus 6.0 was used to analyze immunofluorescence and immunohistochemistry and export measurement data.

### ELISA

Mice serum was collected post-surgery and stored at − 80 °C until required for analysis. The pre-coated plates with specific capture antibodies for IL-6 (EK206HS, Multi Sciences, China), TNF-α (EK282EG, Multi Sciences, China), and LPS (A39552, ThermoFisher, USA) were prepared. Standards for IL-6, TNF-α, and LPS were added to their designated wells, followed by the addition of mouse serum samples into their corresponding wells. The plate was left to incubate for 24 h at room temperature, allowing the target proteins to bind to the capture antibodies. The substrate solution for HRP was then added to each well, and color development was closely monitored for a period ranging from 10 to 30 min. The enzymatic reaction was halted by introducing a stop solution, causing the solution to turn yellow. Absorbance readings were then taken at 450 nm using a microplate reader (BioTek Synergy HTX, Agilent, USA).

### Golgi staining

Fresh brain tissue from mice was soaked in a mixture of reagents A and B from the Rapid GolgiStainTM Kit (PK401, FD NeuroTechnologies, USA) in the dark for 2 weeks. After 2 weeks, the mouse brain tissue was transferred to Reagent C and immersed in the dark for 72 h (reagent C was replaced every 24–48 h). Mouse brain tissue was snap-frozen, and the frozen brain tissue was mounted on a cryostat (CM1950, LEICA, Germany) holder using OCT (1794–00, Sakura Finetek, USA) and cut inside the cryostat at − 22 °C into 100-μM-thick brain slices. Slides were smeared with Reagent C, and the brain sections were transferred onto glass slides. The working solution of the staining solution was prepared using reagents D and E and water, and stained for 10 min. Ultrapure water was used to wash off excess staining solution, and the brain tissue was incubated with 50, 75, 95, and 100% ethanol for 4 min to dehydrate the brain in a gradient. Afterwards, the slides were mounted with neutral resin (10,004,160, Sinopharm Chemical Reagent Co., Ltd., China), observed, and photographed under a microscope (U-RFL-T, Olympus, Japan).

### Detection of gut microbiota by 16S rRNA gene sequencing

After the feces of the mice were collected, genomic DNA was extracted using a fecal DNA extraction kit (Qiagen, Hilden, Germany) according to the manufacturer’s instructions, and the concentration, purity, and integrity of the fecal DNA of each group of mice were determined. Using the extracted DNA as a template, the DNA was amplified using the forward primer (5′-CCTACGGGGNGGCWGCAG3′) and reverse primer (5′-GACTACHVGGGTATCTAATCC-3′). The 5′ end of the primers included the universal sequence of the Illumina adapter. The obtained PCR products were purified and introduced into specific tag sequences compatible with the Illumina platform using high-fidelity PCR to construct the final complete library. After the library quality was assessed, the V3–V4 hypervariable region of the 16S rRNA gene was sequenced using an Illumina MiSeq sequencer to generate raw reads. Quality-filtered and merged the raw reads were merged and quality-filtered. The above steps were performed by Nanjing Paisenuo Gene Technology Co., Ltd. (Nanjing, China). Then, using the GENESCLOUD (https://www.genescloud.cn/home) online analysis tool, the α-diversity, β-diversity, and composition of intestinal flora were analyzed.

### Intestinal permeability detection

To access in vivo intestinal permeability, 600 mg/kg of FITC-dextran (wt 4000; Sigma-Aldrich) was orally given to mice 4 h prior to putting them into a small animal multispectral in vivo imaging system (PerkinElmer IVIS Spectrum). Mice were anesthetized and abdominal hair was removed. The different groups of mice were placed together in prone position in the recording dark box of the small animal multispectral in vivo imaging system, and the mice without FITC-dextran gavage were used as controls. The exposure time was 500 ms, and the images of the animals emitting fluorescence in vivo were recorded. Blood was collected from the mice immediately after imaging. A standard curve was plotted using FITC- dextran as a standard, serum FITC-dextran fluorescence intensity was measured by using a fluorescence microplate reader (Thermo Scientific™ Varioskan™ LUX).

### Measurement of intestinal blood flow

Intestinal blood flow was measured using a laser Doppler flowmeter. A low-power laser beam was directed to the exposed intestine using a computer-controlled optical scanner. The mouse was placed on a thermostatic table, the abdominal cavity was opened, and the scanner head was placed parallel to the exposed intestine at a distance of approximately 20 cm. Subsequently, a color-coded image representing a specific relative perfusion level was displayed on a video monitor. Blood flow values were recorded and measured using the Moor FLPIR V40 program (Gene and I Science. Ltd.).

### Non-targeted metabolomics in mouse serum

The serum from each group of mice was collected, and the serum (50 μL) or standard substance was added to a 1.5-mL centrifuge tube and mixed thoroughly with 200 μL of ice-cold methanol solution (containing 12.5 μg/ml of 1,2-13C myristic acid). The mixture was then centrifuged at 18,000 rpm for 10 min. One hundred microliters of the sample’s supernatant was evaporated using a centrifugal concentrator. Next, 30 μL of methoxyamine pyridine (10 mg/mL) solution was added to the evaporated sample tube, incubated with constant temperature shaking for 1.5 h, 30 μL of BSTFA was added, mixed vigorously, and incubated with constant temperature shaking for 0.5 h. After centrifugation at 18,000 rpm for 10 min, 50 μL of supernatant was collected and placed in a sample vial.

UHPLC analysis was performed on a 1290 LC system (Agilent Technologies) and chromatographic separation was performed on a Waters HSS T3 C18 column (2.1 mm × 100 mm, 1.8 μm). The mobile phases of 0.1% formic acid (A) and acetonitrile (B) were used. The initial mobile phase was 98% B, which was then changed to 92% B for 2 min, 30% B for 6 min, 5% B for 7 min, and 98% B for 0.1 min. The flow rate was 0.3 mL/min.

#### UHPLC-Q-TOF–MS conditions

The UHPLC system was connected to a 6538 Q-TOF (Agilent Technologies) equipped with an electrospray interface operating in positive and negative ion modes under the following conditions: capillary voltage, 4000 V; nebulizer pressure, 40 psi; flow rate of drying gas, 10 L/min; gas temperature, 300 °C; skimmer voltage, 50 V; octopole radiofrequency, 150 V; and fragmentor voltage, 130 V. Accurate LC–MS mass spectra were recorded across an m/z range of 100–1000 Da.

After annotation was completed, a small-scale database with substance names, retention times, and accurate mass-to-charge ratios (m/z) was established. Further statistical analyses were performed using the raw data files. Peak areas of raw metabolites were partially analyzed using MetaboAnalyst 5.0 (https://www.metaboanalyst.ca/MetaboAnalyst/ModuleView.xhtml). Data normalization, complementation, and log calculations were performed using MetaboAnalyst 5.0, and PCA and OPLS analyses were performed for all metabolites. The screening conditions for the differential metabolites were set at *P* ≤ 0.05. Differential metabolites were subjected to enrichment analysis using the MetaboAnalyst 5.0. Vigorous heatmaps of the serum fractionated off-target metabolites were also drawn using MetaboAnalyst 5.0.

### UHPLC detects hippocampus and serum PA levels

Sample processing method: for serum, 100 μL of mouse serum was precisely aspirated in a 1.5-mL EP tube with 400 μL of methanol; vortexed for 3 min, centrifuged at 12,000 rpm × 5 min, 4 ℃; 200 μL of the supernatant was aspirated in another 1.5-mL EP tube; the new tube was centrifuged at 12,000 rpm × 5 min, 4 ℃; 50 μL of the supernatant was aspirated in a liner tube for sampling. For brain tissue, precisely weighing 50 mg of mouse brain tissue (hippocampus), adding 1 ml of cold pure water and homogenizing. Four hundred microliters of pre-cooled methanol was added, vortexed for 3 min, centrifuged at 12,000 rpm for 5 min at 4 ℃; 200 μL of the supernatant was aspirated in another 1.5-mL EP tube; the new tube was centrifuged at 12,000 rpm × 5 min, 4 ℃; 50 μL of the supernatant was aspirated in a liner tube for sampling. For standard substance, diluted PA with methanol to make the final concentrations of PA were 6.25, 12.5, 25.0, 50.0, 100.0, and 200.0 ng/mL.

UHPLC analysis was performed on a 1290 LC system (Agilent Technologies) and chromatographic separation was performed on an ACE Excel 3 C18 (2.1 mm × 100 mm, 1.8 µm); mobile phase acetonitrile (A)-0.2% formic acid water (B) = 70:30 was used, isocratic elution; flow rate was 200 μL/min; column temperature was 35 ℃; injection volume was 10 μL. m/z 256.2 → 88.1 (Fragmentor 150 V, CE 22 V) was PA. The PA levels in mouse hippocampal tissue and serum were calculated by the standard curve.

### Statistical analysis

The gut microbiota and metabolomic data were analyzed using platform analysis tools and analysis methods. Other data are expressed as mean ± standard deviation (mean ± SD), and GraphPad software (version 9.0; Company, USA) was used for the statistical analysis and data visualization. One-way or two-way ANOVA and Dunn’s multiple comparison tests were used to compare the four groups. Differences between the two groups were compared using the Mann–Whitney test. Differences were considered statistically significant at *P* < 0.05.

## Results

### Surgery induces cognitive dysfunction in aged but not young mice

**Fig. 1:**
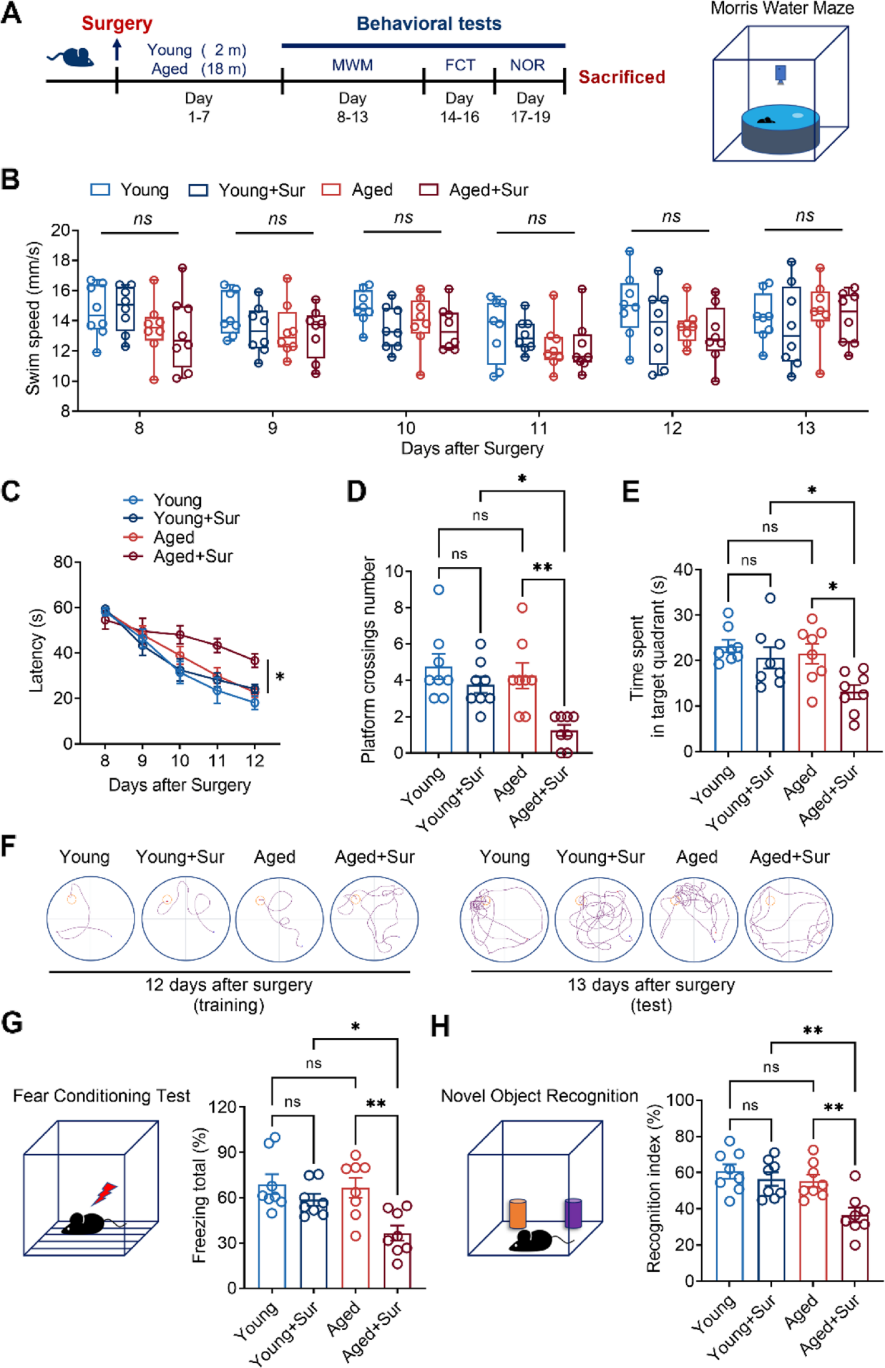
Surgery induces cognitive dysfunction in aged but not young mice. **A** Flow chart of the experiment indicates the age of young and old mice, the time of surgery, and the detection time of each cognitive behavior. **B** The upper right panel is a schematic diagram of the MWM test, and the lower image shows the swimming speed of mice in each group from 8 to 13 days after surgery. **C** Latency of mice in each group to find the platform in the MWM test from 8 to 12 days after surgery. **D** The number of times of mice in each group crossed the platform in the MWM test in the 13th day after surgery. **E** The retention time of mice in each group in the platform quadrant in the MWM test in the 13th day after surgery. **F** Swimming path diagrams of mice in each group in the MWM test on postoperative day 12 (left) and day 13 (right). **G** Schematic diagram of the fear conditioning test (left) and freezing time of mice in each group in the fear conditioning test in the 16th day after surgery (right). **H** Schematic diagram of the novel object recognition test (left) and novel object exploration index in novel object recognition test of mice in each group in the 19th days after surgery (right). *P < 0.05, **P < 0.01, ns means no significant

First, young (2-month-old) or aged (18-month-old) mice were subjected to exploratory laparotomy, and young and aged mice without surgery were used as controls. Seven days after the operation, the cognitive behavior of the mice was examined using MWM, FCT, and NORT. The experimental flowchart indicates the time points at which the mice were manipulated (Fig. 1A). During the experiment, the body weight and food intake of the mice were recorded. Whether in young or aged mice, there were no changes in body weight and food intake within the 2 weeks following the laparotomy (Supplementary Figure S1). The MWM results showed that laparotomy did not cause dyskinesia in young or aged mice. The swimming speed of the mice was tested for five consecutive days from day 8 to day 13 post-surgery. There was no significant difference in the swimming speed between the groups (Fig. 1B). Compared to young mice, there was no significant difference in escape latency in aged mice. After surgery, only the aged mice had a significant increase in the escape latency (Fig. 1C). In the probe test 13 days after the surgery, only the aged mice showed impaired memory, manifested by a decrease in the number of platform crossings and the time spent in the platform quadrant (Fig. 1D–F). In the subsequent FCT, the postoperative freezing time of aged mice decreased significantly, whereas the postoperative freezing time of young mice only slightly decreased (Fig. 1G). Similarly, in the NORT, young or aged mice without surgery spent more time exploring new objects and showed a higher recognition index, indicating that they had memories of familiar objects. In contrast, the recognition index of aged mice was significantly reduced, while that of young mice was only slightly decrease (Fig. 1H). These results suggest that surgery leads to cognitive impairment in aged but not young mice.

### Surgery causes brain pathological changes in aged mice

**Fig. 2:**
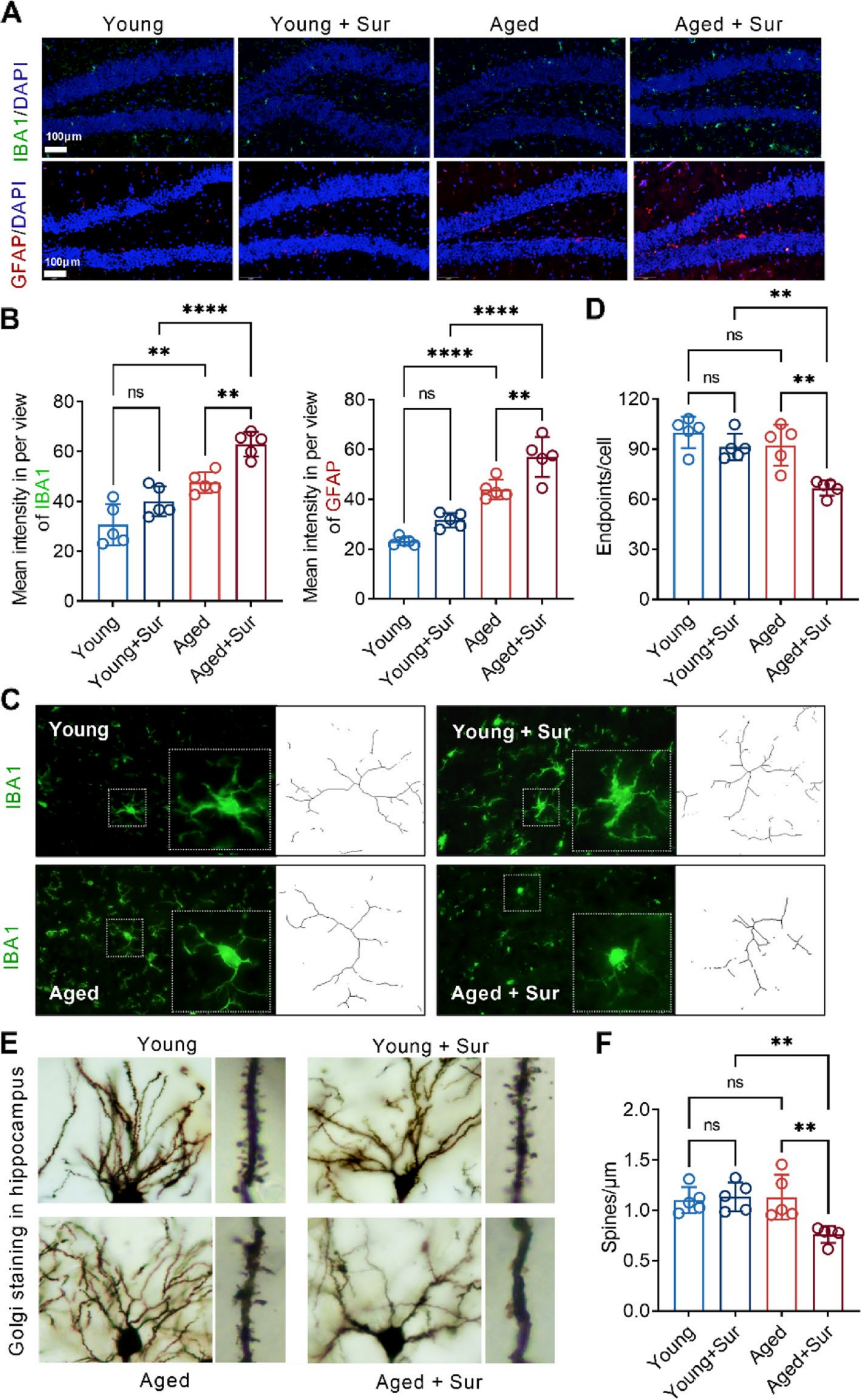
Surgery causes brain pathological changes in aged mice. **A** Immunofluorescence staining was used to detect the expression level of the microglial marker IBA1 (green) and astrocyte marker GFAP (red) in the hippocampus of mouse brain tissues in each group. **B** Correlation with **A**, the fluorescence intensity of IBA1 and GFAP in the hippocampus of the mouse brain tissues in each group was calculated. **C** Confocal laser microscopy magnified the microglia positively expressing IBA1 in the hippocampus of mouse brain tissues in each group. **D** Correlation with **C**, the number of branches of microglia expressing IBA1 positively in the hippocampus of the mouse brain tissues of each group was calculated. **E** Golgi staining was used to detect morphological changes in neurons and dendrites in the hippocampus of mice in each group. **F** Correlation with **E**, the length of the spines in each group of mouse neurons under the same magnification. **P < 0.01, ****P < 0.0001, ns means no significant

After the 2- and 18-month-old mice underwent surgery and completed behavioral tests, brain tissues were obtained, and histological examinations were performed on the 20th postoperative day. Immunofluorescence staining showed that the surgery resulted in significant activation of the microglia in the hippocampus of aged mouse brain tissue, which was manifested by an increase in the number and intensity of IBA1 fluorescence-positive cells. The results of immunofluorescence staining also showed that the surgery led to significant activation of astrocytes in the hippocampus of the aged mouse, which was manifested by an increase in the number and intensity of GFAP fluorescence-positive cells. These changes in IBA1 and GFAP were not observed in the hippocampal region of the brain tissue of young mice underwent surgery (Fig. 2A and B). Surgery caused the microglia in the hippocampus of aged mice to transform from a complex morphology with small nuclei and long protrusions to an activated morphology, which turned into enlarged and thickened cell bodies with reduced branching. However, these changes were not observed in the hippocampus of young mice underwent surgery (Fig. 2C and D). Golgi staining showed that the number of ganglia in the dendrites and axons of the aged mice decreased after the operation, while surgery had no significant effect on the neurons of the young mice (Fig. 2E and F). These results indicate that neuroinflammation and loss of dendritic spines caused by surgery are more severe in the aged mice.

### Aged mice exhibit gut microbial dysbiosis, and exacerbated by surgery

**Fig. 3:**
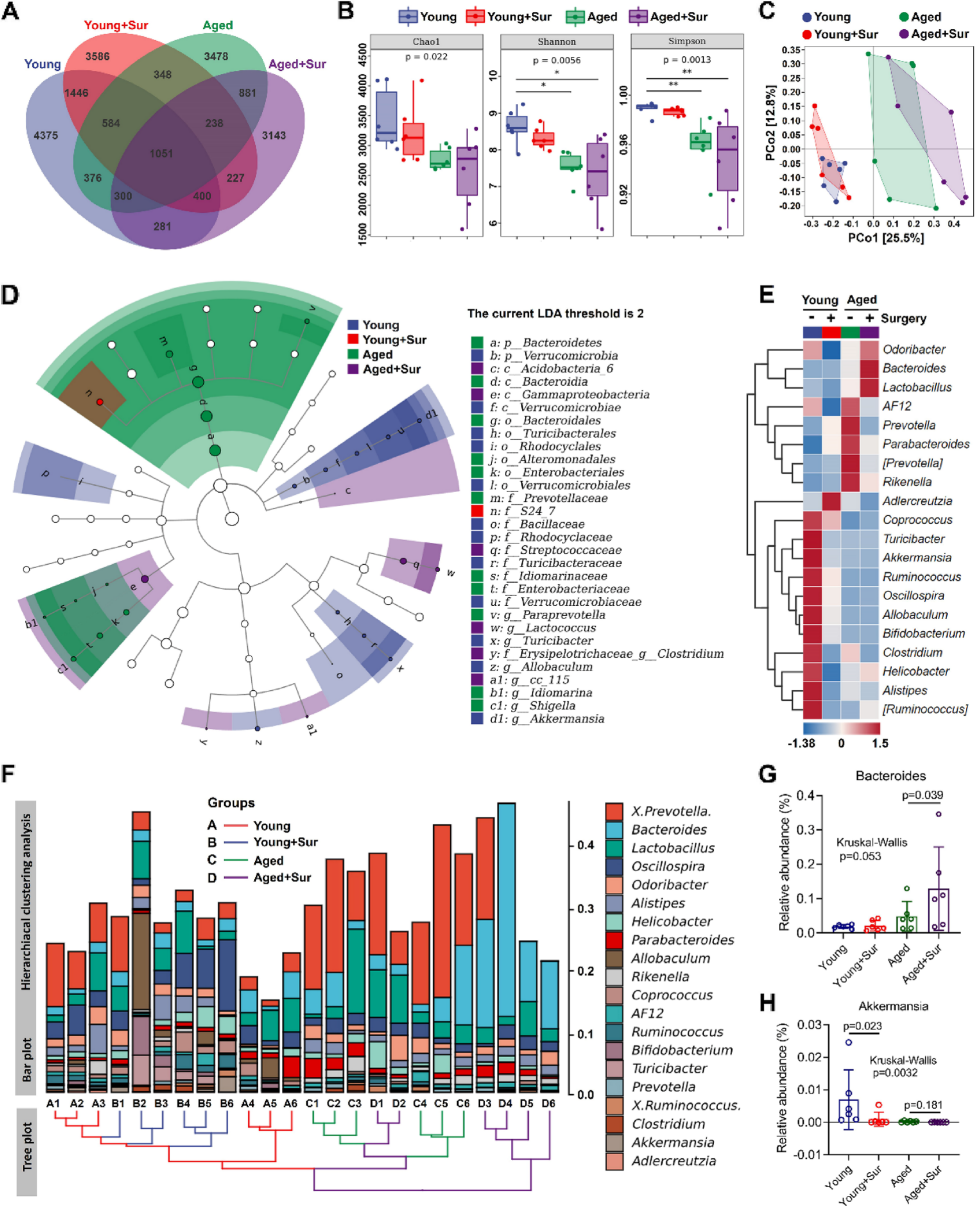
Aged mice exhibit gut microbial dysbiosis, and exacerbated by surgery. **A** Venn diagram indicates the OTUs detected in the feces by 16S rRNA gene sequencing of four groups of mice, as well as shows OTUs unique to each group and OTUs share among different groups. **B** Analysis of the α-diversity of gut microbiota in the feces of the four groups of mice. **C** Principal coordinates analysis (PCoA) was used to calculate the Bray–Curtis distance matrix to analyze the beta diversity of gut microbiota in the feces of four groups of mice. **D** Linear discriminant analysis Effect Size (LEfSe) was used to find species with significant differences at all taxonomic levels of the gut microbiota in the feces of four groups of mice. The comparison strategy was one-against-all, and the LDA threshold was 2. **E** Cluster analysis heatmap of genus-level species composition of the feces indicates the relative abundance changes of the top 20 genus-level flora species (average of each group) among the four groups of mice. **F** Hierarchical clustering analysis shows the similarity between the four groups of mouse feces and indicates the composition of species at the genus level (top 20) in every single sample. **G** The abundance values of *g_Bacteroidetes* in the four groups were extracted from the species abundance table and the differences between the four groups were analyzed. **H** The *g_Akkermansia* abundance values in the four groups were extracted from the species abundance table and the differences between the four groups were analyzed

To identify specific changes in the gut microbiota with surgery, we performed 16S rRNA gene sequencing of mice that underwent surgery. Detection of the microbial profile in the feces of young or aged mice with or without surgery showed that the number of operational taxonomic units (OTUs) detected in the feces of aged mice was lower than that in young mice. In both young and aged mice, surgery reduced the number of OTUs detected in feces (Fig. 3A). The results of the α-diversity analysis show that the abundance (Chao1) and diversity (Shannon and Simpson) of the gut microbiota in the feces of aged mice were significantly lower than those of young mice. Consistent with the number of OTUs, surgery reduced the diversity of the gut microbiota in the feces of young and aged mice (Fig. 3B). PCoA was used to analyze the β-diversity of the gut microbiota in the feces, and the results showed that the overall species composition of young and aged mice was clearly separated. In aged mice, surgery resulted in significant separation in the β-diversity of gut microbiota (Fig. 3C). Linear discriminant analysis Effect Size (LEfSe) was used to analyze the differences in all the classification levels of the gut microbiota in the feces to identify the different species between young and aged mice and between non-surgical and surgical mice. The results showed that *p_Verrucomicrobia* in the feces of young mice (blue) were significantly different from that in the feces of the other groups. The *p_Bacteroidetes* in the feces of aged mice (green) was significantly different from those of the other groups. *f_S24_7* in the feces of Young + Sur mice (red) was significantly different from that of the other groups. The abundance of *f. Streptococcaceae* in the feces of Aged + Sur (purple) mice was significantly different from that in the feces of other groups (Fig. 3D). To further clarify the changes in the composition of the gut microbiota, cluster heatmaps were used to show the relative levels of the genus-level (Top 20) gut microbiota in the feces of the four groups (Fig. 3E). At the same time, the relative level of the gut microbiota at the genus level (Top 20) in the feces of each mouse is shown in a clustered histogram (Fig. 3F). The results showed that, compared with young mice, the level of *g_Bacteroidetes* in the feces of aged mice increased significantly, while the level of *g_Akkermansia* (the main species of *p_Verrucomicrobia*) decreased drastically (Fig. 3E–H). In young mice, surgery did not cause changes in *p_Bacteroidetes*; however, in aged mice, surgery increased the level of *p_Bacteroidetes* in feces (Fig. 3G). In young mice, surgery resulted in a significant decrease in *g_Akkermansia* levels. Although there was no significant difference between the level of *g_Akkermansia* in the feces of aged mice and the feces of non-operated aged mice, this might be attributed to the very low *g_Akkermansia* levels in feces of aged mice (Fig. 3H). These results indicate significant differences in the composition of the gut microbiota of young and aged mice and that the surgery caused significant changes in the species diversity of the gut microbiota and the microbiota of specific species, whether in young or aged mice.

To substantiate the findings detailed and to ascertain that the behavioral tests did not skew the gut microbiota observations, we mirrored the original research design. A meticulous examination of gut microbiota variations in both young and aged mice, pre- and post-behavioral tests, was undertaken (Supplementary Figure S2). Supplementary Figure S2A, presenting a bar graph of microbial species composition, suggests that young mice maintain a consistent gut microbiota profile, largely undisturbed by both surgery and behavioral tests. In contrast, aged mice exhibited a pronounced shift in their microbial species diversity, especially following surgery. This distinction is further illuminated in Supplementary Figure S2B, where the Venn diagram showcases the absolute counts of the entire microbial community detected across various groups. Here, surgery emerged as a potent factor diminishing the total microbial count in both age cohorts. However, an intriguing observation was the resilience shown by the young mice post-surgery, even after undergoing behavioral tests, maintaining a relatively steady microbial count. On the flip side, while aged mice post-surgery showed a decline, their microbial count did not drop further post-behavioral tests but saw a marginal increase (Supplementary Figure S2A-B). The α-diversity analysis depicted in Supplementary Figure S2C mirrored the findings from Supplementary Figure S2A-B. Yet, aged mice did undergo marked β-diversity shifts, as evidenced in the NMDS and substantiated by the PERMANOVA findings, but changed little by behavioral tests (Supplementary Figure S2D-E). The LEfSe analysis further corroborated the earlier observations (Fig. 3D–H and Supplementary Figure S2F), emphasizing the significant bacterial taxa discrepancies between the young and aged mice, a pattern that endured irrespective of behavioral interventions. In summing up, the aged mice, with their already divergent microbial blueprint, showcase enhanced vulnerability to these external stimuli, especially surgical procedures.

### Surgery impaired gut barrier in aged but not young mice

**Fig. 4:**
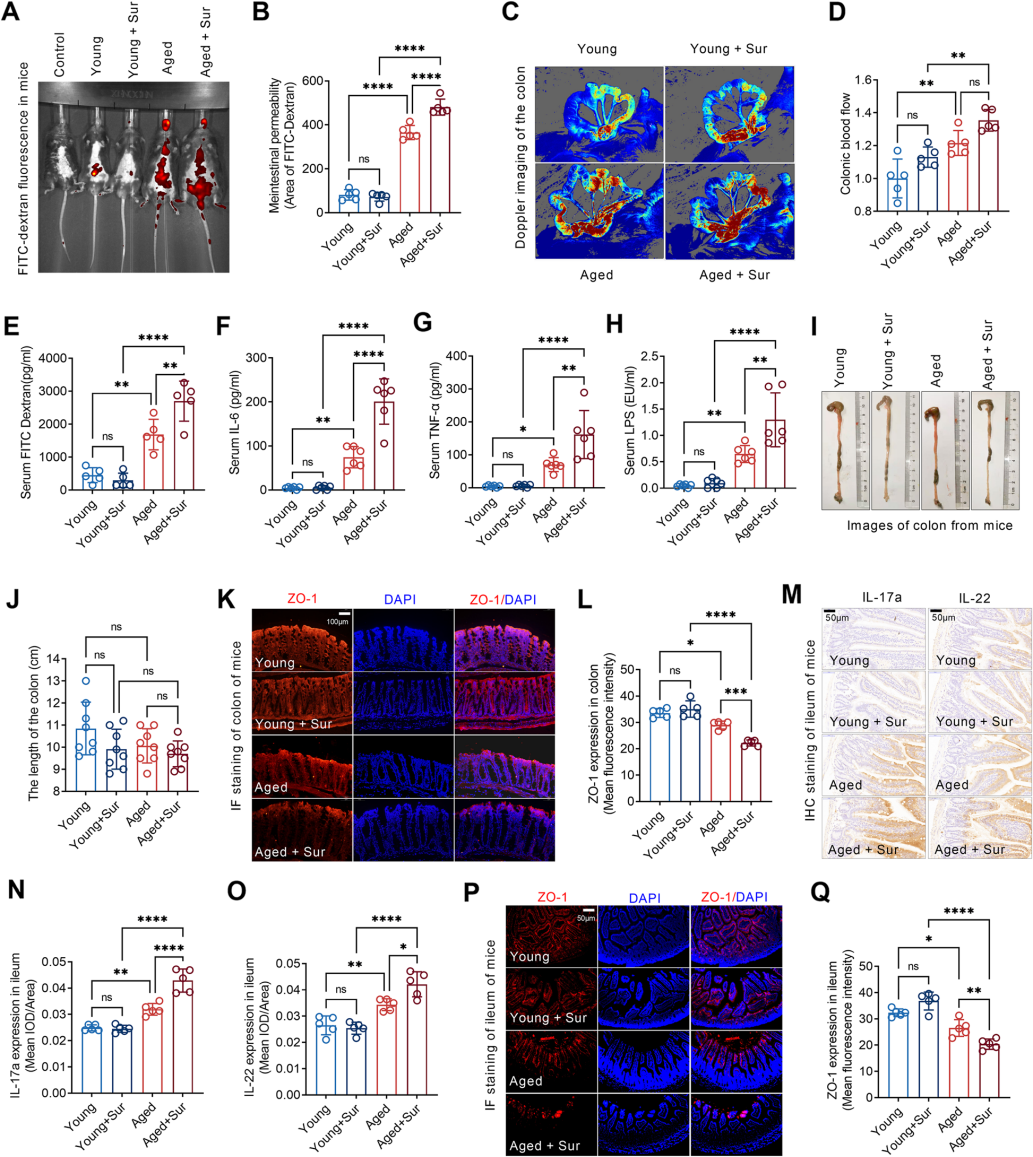
Surgery impaired gut barrier in aged but not young mice. **A** VISQUE® InVivo Smart-LF system was used to image the mice with FITC-dextran gavage treatment, no FITC-dextran gavage mice were used as control. The red area displays the fluorescence-positive area of FITC-dextran after enhancement. **B** Correlation with **A**, positive area of FITC-dextran in mice was calculated using ImageJ to indicate the permeability of the mouse intestine. **C** Mice were exposed to the abdominal colon before sacrifice and the colon was imaged using the mouse Doppler system. **D** Correlation with **C**, quantitative statistics of colonic blood flow in mice using the Moor FLPIR V40 program. **E** Levels of FITC-dextran in mouse serum were measured using a multifunctional fluorescence detector after 4 h of FITC-dextran gavage. **F** ELISA was used to detect the IL-6 level in the mouse serum in each group. **G** ELISA was used to detect the TNF-α level in the mouse serum in each group. **H** ELISA was used to detect the LPS level in the mouse serum in each group. **I** Representative images of the cecum and colon of each group of mice, with a straightedge indicating the length of the colon. **J** Correlation with **I**, the differences between the four groups of the length of colon were analyzed. **K** Immunofluorescence staining was used to detect the expression level of the tight junction protein ZO-1 (red) in the mouse colon tissues in each group. **L** Correlation with **K**, the fluorescence intensity of ZO-1 in the mouse colon tissues was calculated. **M** Immunohistochemical staining was used to detect the expression level of IL-17a and IL-22 in the mouse ileum tissues in each group. **N** Correlation with **M**, the mean IOD/area of IL-17a in ileum was calculated. **O** Correlation with **M**, the mean IOD/area of IL-22 in ileum was calculated. **P** Immunofluorescence staining was used to detects the expression level of the tight junction protein ZO-1 (red) in the mouse ileum tissues in each group. **Q** Correlation with **P**, the fluorescence intensity of ZO-1 in the mouse ileum tissues was calculated. *P < 0.05, **P < 0.01, ***P < 0.001, ****P < 0.0001, ns means no significant

Recent studies have demonstrated that impaired gut barrier has been associated with multiple neurological diseases, so-called “leaky gut” [17, 18]. Therefore, we investigated intestinal permeability in the mice to determine how surgery-induced microbial dysbiosis contributes to PND. This variation is evident when examining the FITC-dextran-induced fluorescence within the mice. Utilizing the VISQUE® InVivo Smart-LF system, we visualized enhanced fluorescence in the treated mice, compared to controls without FITC-dextran gavage (Fig. 4A). Analysis of this fluorescence, indicative of intestinal permeability, was quantified using ImageJ. We observed a prominent increase in FITC-dextran fluorescence in aged mice post-surgery, which was not present in their younger counterparts (Fig. 4B). Prior to sacrifice, the abdominal colon of mice was exposed, and images captured using a Doppler system portrayed no discernible variation in colonic blood flow between mice that underwent surgery and those that did not, as quantified using the Moor FLPIR V40 program (Fig. 4C and D). Additionally, the levels of FITC-dextran in the serum, measured post 4-h gavage using a fluorescence detector, showed a heightened presence in aged mice after surgery. In contrast, the young post-surgery mice displayed no such amplification (Fig. 4E). Subsequent ELISA tests of the mouse serum revealed noticeable elevations in IL-6, TNF-α, and LPS levels in the aged mice post-surgery, compared to other groups (Fig. 4F–H). Further examination of the cecum and colon, illustrated by representative images, highlighted no significant differences in the colon lengths across all groups (Fig. 4I and J). The tight junction-associated protein ZO-1 plays a pivotal role in maintaining gut barrier function. Immunofluorescence staining showed a marked reduction of ZO-1 expression in the colons of aged mice after surgery, a contrast to young mice that remained unaffected (Fig. 4K and L). In the mouse ileum tissues, immunohistochemical staining indicated the expression of IL-17a and IL-22. The IOD intensities for both these cytokines were discernibly higher in aged mice post-surgery, providing further insight into the impact of surgical interventions on aged mice gut integrity (Fig. 4M–O). Finally, the examination of ZO-1 expression in the ileum tissues, again via immunofluorescence staining, mirrored the findings from the colon, with a substantial decline noted in aged mice post-surgery (Fig. 4P–Q). In summary, these observations emphasize that while surgery affects the gut barrier in aged mice, largely by downregulating tight junction proteins and altering inflammatory cytokine levels, young mice seem resilient to such surgical-induced changes.

### FMT or anti-inflammatory agent reverses surgery-associated breakdown of gut barrier integrity and alleviates PND

**Fig. 5:**
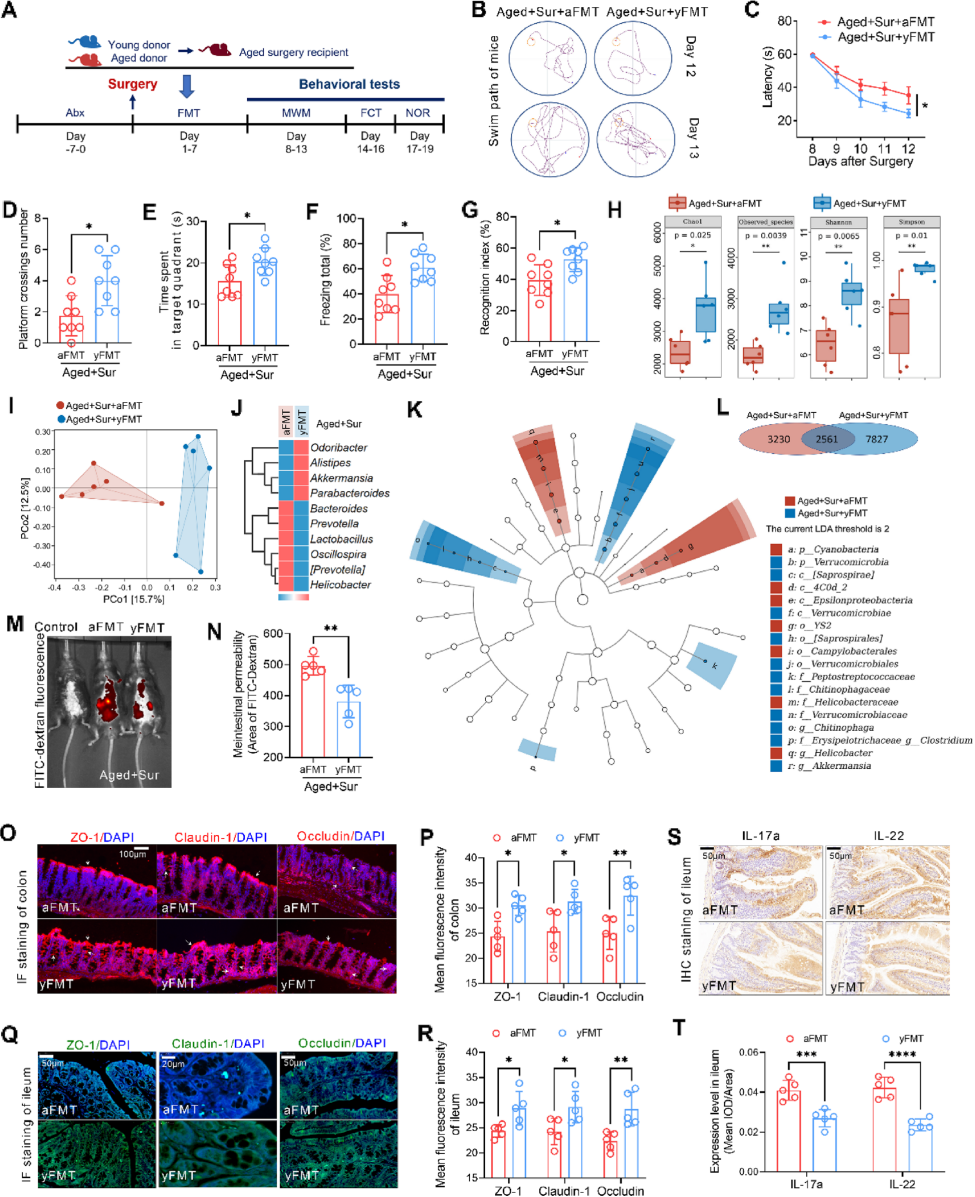
Remodeling of gut microbiota by FMT reverses surgery-associated breakdown of gut barrier integrity and alleviates PND. **A** Flow chart of the experiment indicates the handling of the donor and recipient mice in the FMT experiment and the detection time of each cognitive behavior. **B** Swimming path diagrams of the two groups of mice in the MWM test on postoperative day 12 and day 13. **C** Latency to find the platform in the MWM test from 8 to 12 days after surgery of the two groups of mice. **D** The retention time of the two groups of mice in the platform quadrant in the MWM test in the 13th day after surgery. **E** The number of times that the two groups of mice crossed the platform in the MWM test in the 13th day after surgery. **F** Freezing time in the fear conditioning test in the 16th day after surgery of the two groups of mice. **G** Novel object exploration index in novel object recognition in the 19th day after surgery in both groups of mice. **H** Analysis of the α-diversity of the gut microbiota in the feces of the two groups of mice. **I** PCoA was used to calculate the Bray–Curtis distance matrix to analyze the beta diversity of gut microbiota in the feces of the two groups of mice. **J** Cluster analysis heatmap of genus-level species composition of the feces indicates the relative abundance changes of the top 10 genus-level flora species (average of each group) among the two groups of mice. **K** LEfSe was used to find species with significant differences at all taxonomic levels of the gut microbiota in the feces of the two groups of mice. The comparison strategy was one-against-all, and the LDA threshold was 2. **L** Venn diagram indicates the OTUs detected in the feces by 16S rRNA gene sequencing, as well as shows OTUs unique to each group and OTUs share among the two groups. **M** In vivo imaging system was used to image the mice with FITC-dextran gavage treatment in the two groups, no FITC-dextran gavage mice were used as control. The red area displays the fluorescence-positive area of FITC-dextran after enhancement. **N** Correlation with **M**, positive area of FITC-dextran in mice of the two groups was calculated using ImageJ to indicate the permeability of the intestine. **O** Immunofluorescence staining was used to detect the expression level of the tight junction protein ZO-1, Cluadin-1, and Occludin in the mouse colon tissues in the two groups. **P** Correlation with O, the fluorescence intensity of ZO-1, Cluadin-1, and Occludin in the mouse colon tissues in the two groups was calculated. **Q** Immunofluorescence staining was used to detect the expression level of the tight junction protein ZO-1, Cluadin-1, and Occludin in the mouse ileum tissues in the two groups. **R** Correlation with **Q**, the fluorescence intensity of ZO-1, Cluadin-1, and Occludin in the mouse ileum tissues in the two groups was calculated. **S** Immunohistochemical staining was used to detect the expression level of IL-17a and IL-22 in the mouse ileum tissues in the two groups. **T** Correlation with **R**, the mean IOD/area of IL-17a and IL-22 in ileum was calculated. *P < 0.05, **P < 0.01, ***P < 0.001, ****P < 0.0001

To directly test the effect of gut microbiota on PND, fecal bacterial transplantation (FMT) was performed on aged mice underwent surgery, and the donor mice were young and aged mice. After undergoing laparotomy, aged mice immediately received FMT from young (2 months old) or aged (18 months old) mice for 7 days. The experimental flowchart indicates the time points at which the mice were manipulated (Fig. 5A). Prior to receiving FMT, all recipient mice were treated with antibiotics, which significantly suppressed the overall species level of their gut microbiota, as evidenced in Supplementary Figure S3. The synergistic application of vancomycin and ampicillin exhibits a profound suppressive impact on the gut microbiota of mice, regardless of their age. Both the overall microbial count (Supplementary Figure S3A-B) and microbial α- and β-diversity (Supplementary Figure S3C-E) witness substantial reductions, highlighting the potency and broad-spectrum efficacy of the antibiotic regimen employed. The results of the MWM showed that compared to mice that received aged mice feces, mice that received young mouse feces had significantly decreased postoperative escape latency and increased the platform crossing time and the time spent in the platform quadrant (Fig. 5B–E). In the FCT, the postoperative freezing time of the mice that received feces from young mice increased significantly (Fig. 5F). Similarly, in the NORT, the age surgery mice that received young mice feces spent more time exploring new objects and showed a higher recognition index (Fig. 5G). These results suggest that FMT ameliorated surgery-induced cognitive impairment in aged mice.

Furthermore, compared with FMT from aged mice, FMT from young mice increased the overall abundance (Chao and Observed_species) and diversity (Shannon and Simpson) of gut microbes (Fig. 5H). PCoA results showed that the FMT from young mice increased the β-diversity of the gut microbiota in the feces (Fig. 5I). To further clarify the changes in the composition of the gut microbiota after FMT, the relative levels of the genus level (Top10) of gut microbiota were shown using cluster heatmaps. The results showed that compared with the FMT from aged mice, the level of *g_Akkermansia* in the feces of the FMT from young mice were increased significantly, while the level of *g_Bacteroidetes* were decreased (Fig. 5J). LEfSe analysis show that *p_Verrucomicrobia* significantly changed in the feces of FMT from young mice (blue). The *p_Cyanobacteria* in the feces of FMT from aged mice (red) significantly changed (Fig. 5K). The results of Venn diagram analysis show that, compared with the FMT from aged mice, the FMT from young mice increased the number of detected OTUs in the feces (Fig. 5L). The FITC- dextran was used to investigated the intestinal permeability. Compared to FMT from aged mice, significant decrease in intestinal permeability was observed in aged mice underwent surgery receiving FMT from young mice (Fig. 5M–N). We also assessed the colonic tight junction expression in aged mice receiving FMT. FMT from young mice increased the level of ZO-1, claudin-1, and occludin in the aged mice underwent surgery (Fig. 5O and P). Similarly, FMT from young mice significantly upregulated the expression levels of ZO-1, claudin-1, and occludin in the ileum tissues of aged mice post-surgery (Fig. 5Q and R). Moreover, immunohistochemical staining illustrated that FMT from young mice notably attenuated the postoperative upregulation of IL-17a and IL-22 in aged mice (Fig. 5S and T). These findings emphasize the role of FMT in restoring gut barrier functions and downregulating inflammation-associated cytokines in the aged mice subjected to surgery.

The beneficial impact of FMT on PND prompted us to explore the effects of the anti-inflammatory agent, dexamethasone, in a similar context. In MWM, treated aged mice demonstrated reduced latency to find the platform, increased platform crossings, and extended retention time in the platform quadrant (Supplementary Figure S4B-E). Furthermore, the FCT revealed an increased freezing time, and the NORT displayed a higher recognition index in mice treated with dexamethasone (Supplementary Figure S4F-G). Dexamethasone appeared to mitigate surgery-induced neuroinflammation, as indicated by the presence of markers IBA1 and GFAP (Supplementary Figure S4H-I). In the realm of gut barrier integrity, dexamethasone enhanced the expression of tight junction proteins like ZO-1, Cluadin-1, and Occludin (Supplementary Figure S4J-M) and modulated the levels of IL-17a and IL-22 in the ileum (Supplementary Figure S4N).

Furthermore, in the context of the gut microbiota, dexamethasone exerted a pronounced modulatory effect on aged mice following surgical interventions. The bar chart showcases how dexamethasone administration significantly amplified the microbial species composition in postoperative aged mice, bringing them to levels observed in age-matched controls without surgery (Supplementary Figure S5A). Similarly, the Venn diagram underscores the heightened absolute microbial count achieved through the influence of dexamethasone (Supplementary Figure S5B). While the alpha diversity analysis reveals that dexamethasone closely restored the microbial community diversity to that of a typical aged mouse (Supplementary Figure S5C), insights into the beta diversity depict a nuanced picture. Despite the restoration in species count and diversity, the microbial community structure still resembled that of surgically treated aged mice (Supplementary Figure S5D-E). The hierarchical clustering presents a compelling observation: samples from surgically treated aged mice and those treated with dexamethasone postoperation clustered closely, indicating similar microbial compositions (Supplementary Figure S5F). LEfSe analysis offers a glimpse into the specific bacterial taxa influenced by surgery and dexamethasone. While surgery manifested pronounced shifts in particular bacterial taxa, dexamethasone treatment did not introduce many distinctive microbial species (Supplementary Figure S5G). This signifies dexamethasone’s primary role in enhancing the overall abundance and alpha diversity in the gut of aged surgical mice, rather than altering the species composition.

### *Lactobacillus* or indole propionic acid attenuates PND by restoring intestinal barrier function in aged mice

**Fig. 6:**
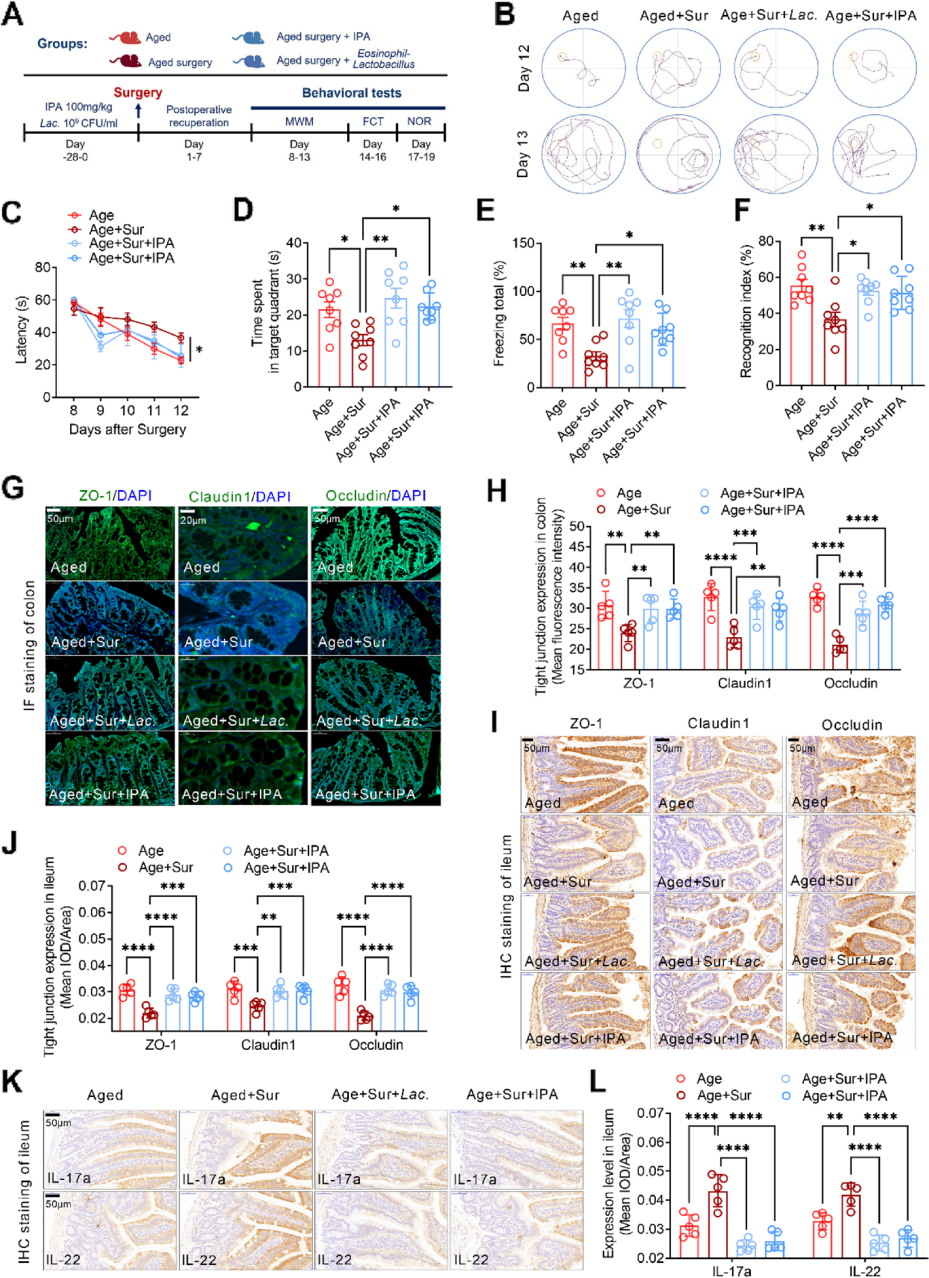
*Lactobacillus* or indole propionic acid attenuates PND by restoring intestinal barrier function in aged mice. **A** Flow chart of the experiment indicates the pretreatment of *Lactobacillus* or IPA in aged mice before surgery and the detection time of each cognitive behavior. **B** Swimming path diagrams of the aged mice with different treatment in the MWM test on postoperative day 12 and day 13. **C** Latency to find the platform in the MWM test from 8 to 12 days after surgery of the aged mice. **D** The retention time in the platform quadrant in the MWM test in the 13th day after surgery of the aged mice. **E** Freezing time in the fear conditioning test in the 16th day after surgery of the aged mice. **F** Novel object exploration index in novel object recognition in the 19th day after surgery of the aged mice. **G** Representative images of immunofluorescence staining of the tight junction protein ZO-1, Cluadin-1, and Occludin in colon tissues of the aged mice with different treatment. **H** Correlation with **G**, the fluorescence intensity of ZO-1, Cluadin-1, and Occludin in the mouse colon tissues was calculated. **I** Representative images of immunohistochemical staining of the tight junction protein ZO-1, Cluadin-1, and Occludin in ileum tissues of the aged mice with different treatment. **J** Correlation with **I**, the mean IOD/area of ZO-1, Cluadin-1, and Occludin in the mouse ileum tissues was calculated. **K** Immunohistochemical staining was used to detect the expression level of IL-17a and IL-22 in ileum tissues of the aged mice with different treatment. **L** Correlation with **K**, the mean IOD/area of IL-17a and IL-22 in ileum was calculated. *P < 0.05, **P < 0.01, ***P < 0.001, ****P < 0.0001

To elucidate the potential therapeutic roles of the intestinal barrier in postoperative neurocognitive disorders (PND), we expanded our approach to include both *Lactobacillus* and indole propionic acid (IPA) treatment, which we have demonstrated its role in improving intestinal barrier in obese mice [14]. A detailed experimental flowchart showcases the pre-surgical administration of either *Lactobacillus* or IPA to aged mice and the subsequent timings of each cognitive assessment (Fig. 6A). Results from the MWM test post-surgery revealed that both *Lactobacillus* and IPA treatments effectively shortened the escape latency and prolonged the time the aged mice spent in the target quadrant, demonstrating enhanced spatial memory and learning (Fig. 6B–D). On postoperative day 16, the FCT showed that these treatments significantly boosted the freezing time, indicating improved associative memory (Fig. 6E). This memory enhancement was further supported by the NORT conducted on day 19 post-surgery, where treated mice exhibited an increased exploration index for new objects, suggesting better recognition and memory retention of familiar objects (Fig. 6F).

Furthering our investigation into the effect of these treatments on the gut barrier, we employed immunofluorescence techniques to assess the levels of essential tight junction proteins. Both *Lactobacillus* and IPA treatments manifested in a significant upregulation of ZO-1, claudin-1, and occludin in the colons of post-surgery aged mice (Fig. 6G and H). A parallel enhancement of these proteins was observed in the ileum tissues of the treated groups (Fig. 6I and J). Immunohistochemical analysis identified elevated levels of IL-17a and IL-22 in untreated mice post-surgery. In contrast, both the *Lactobacillus* and IPA-treated groups displayed subdued levels of these cytokines, emphasizing their potential anti-inflammatory properties (Fig. 6K and L). In summary, the findings emphasize the potential of *Lactobacillus* and IPA treatments in mitigating PND in aged mice, primarily through the enhancement of intestinal barrier function and suppression of inflammatory responses.

### Surgery induces altered serum metabolites and increases PA contents in mice

**Fig. 7:**
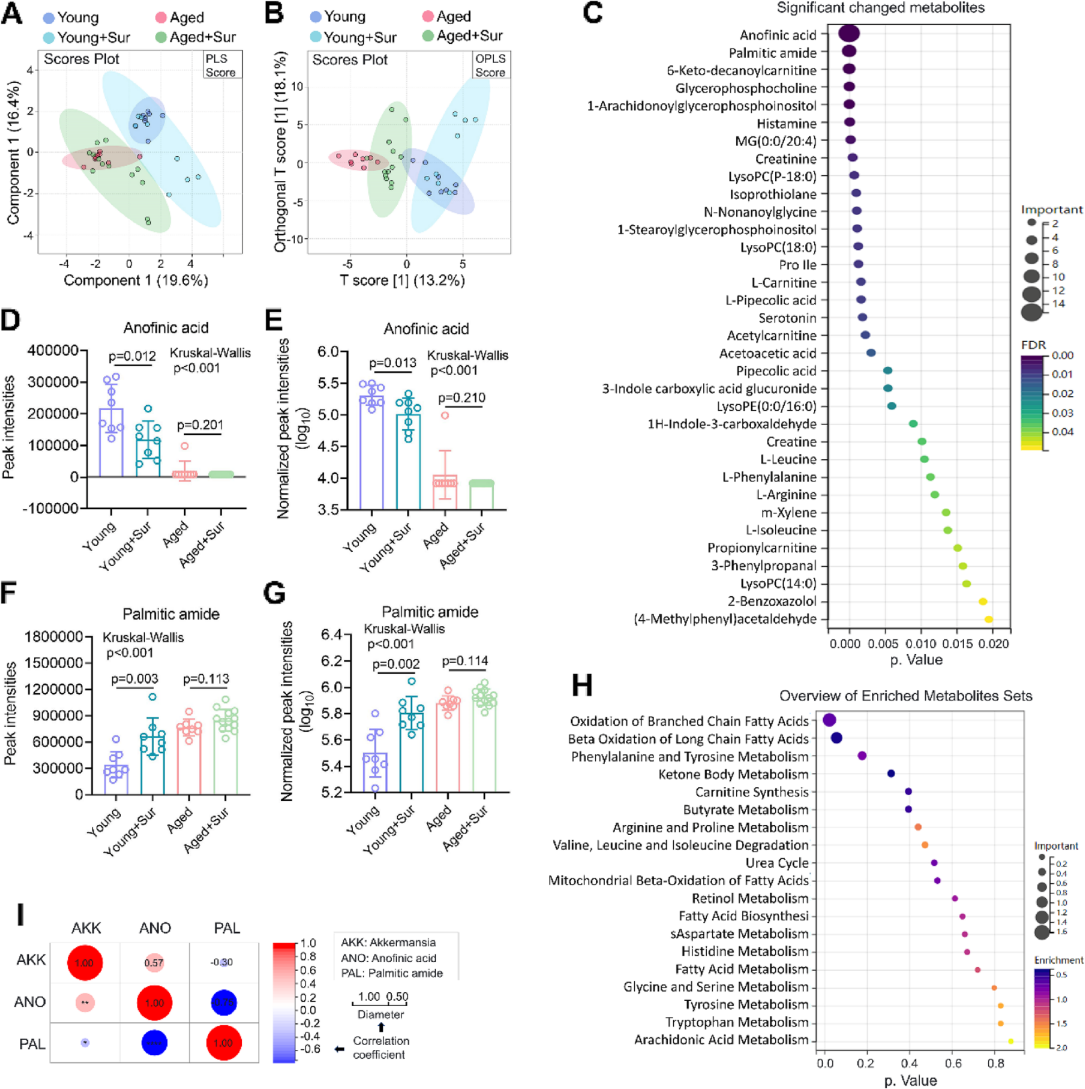
Surgery induces altered serum metabolites and increases PA contents in Mice. **A** Metabolite profiles in the blood of young and aged mice with or without surgery were analyzed by non-target metabolomics. Principal component analysis of all metabolites detected in the blood of four groups of mice by orthogonal partial least squares (PLS). **B** Principal component analysis of all metabolites detected in the blood of four groups of mice by partial least squares (OPLS). **C** Bubble charts indicate the significantly changed differential metabolites in the blood of the four groups of mice, and it indicates the *P* value of each metabolite, the contribution to the difference (importance) and the FDR. **D** Anofinic acid peak areas in each blood sample of four groups of mice were extracted from the metabolic profiles and the differences between groups were analyzed. **E** Anofinic acid peak areas in blood samples from four groups of mice were normalized into logarithmic values and the differences between groups were analyzed. **F** Palmitic amide peak areas in each blood sample of four groups of mice were extracted from the metabolic profiles and the differences between groups were analyzed. **G** Palmitic amide peak areas in blood samples from four groups of mice were normalized into logarithmic values and the differences between groups were analyzed. **H** Metabolic signals were enriched by the significantly changed differential metabolites in the blood of the four groups of mice. The enriched metabolic signals are shown in a bubble chart, and the bubble chart showed all significantly changed metabolic signals. **I** Correlation analysis heatmap of *g_Akkermansia* abundance and levels of anofinic acid and palmitic amide in blood of the four groups of mice. Correlation coefficients are indicated in the heatmap; red represents a positive correlation, blue represents a negative correlation

Modulation of metabolism is an important way in which gut microbes impact central nervous system [19]. Increased intestinal permeability allows metabolites to pass through the intestinal barrier to the bloodstream [20]. To further elucidate the mechanisms how surgery-associated gut microbiota activates PND, we performed an unbiased metabolic screen of the serum of mice. Non-target detection of the metabolite profile in the blood of young or aged mice with or without surgery showed that 86 non-target metabolites were detected and determined in the serum of the mice (available in “figshare”, in “Availability of data and materials” section). Orthogonal partial least squares (OPLS) and partial least squares (PLS) were used to extract load and principal components from the non-target metabolic spectrum of the blood of four groups of mice and then compared between groups. The results showed that the metabolite profiles of young and aged mice were significantly separated, regardless of whether they underwent surgery. Regardless of whether the mice were young or aged, surgery caused changes in the metabolic profile, which was reflected in the increase in dispersion between samples in PLS and OPLS (Fig. 7A and B). Among the detected non-target metabolites (86 kinds), 34 kinds of metabolites showed significant changed between the groups (*P* < 0.05) (Fig. 7C). Among all the metabolites with significant change, anofinic acid and palmitic amide levels changed drastically (Fig. 7C). The changes of them in the blood of the four groups of mice were analyzed separately, and the results showed that when compared with young mice, almost no anofinic acid was detected in the blood of aged mice. In young mice, surgery resulted in a significant decrease in the anofinic acid levels. Additionally, because anofinic acid was almost undetectable in aged mice, there was no significant difference of it between the aged and aged surgical mice (Fig. 7D and E). Palmitic amide increased significantly in aged mice compared with young mice. Surgery further increased its levels in mice, especially in young mice. Although the increase was not significant in aged mice, this may be related to their higher basal level (Fig. 7F and G). Metabolic signal enrichment analysis of the 34 changed metabolites in the blood of the four groups showed that there were 19 metabolism-related signals with significant alterations. Most of these signals were focused on fatty acid metabolism. The metabolism of branched-chain fatty acid oxidation and long-chain fatty acid β-oxidation showed most significantly changed (Fig. 7H). The results of the correlation analysis of *g_Akkermansia* abundance in the feces with the levels of anofinic acid and palmitic amide in the blood showed that the abundance of *g_Akkermansia* and anofinic acid levels were significantly positively correlated, and the abundance of *g_Akkermansia* and palmitic amide levels were negatively correlated (Fig. 7I). This suggests that abnormalities in the gut microbiota resulting from surgery lead to alterations in some specific metabolites in the circulation.

### FMT attenuates PND in aged mice by reversing surgery-associated metabolic perturbations and decreases PA contents

To validate the insights garnered from the FMT experiment and ascertain the extent of protection FMT offered to aged mice against PND in comparison to their younger counterparts, we replicated the FMT research setup. In this design, postoperative young mice received FMT either from young or aged donors (Supplementary Figure S6A). Predictably, mice in the Aged_sur + aFMT group manifested the most compromised behavioral performance (Supplementary Figure S6B-G). This group also showed elevated brain expressions of IBA1 and GFAP (Supplementary Figure S6H-J), as well as heightened gut expressions of IL-17a and IL-2 (Supplementary Figure S6O-P). Moreover, these mice displayed the lowest gut expression levels of tight junction proteins (Supplementary Figure S6K-N). Notably, when young mice were recipients of FMT from older donors, they did not exhibit cognitive dysfunctions or neuroinflammation (Supplementary Figure S6B-J). However, they did show diminished gut expressions of tight junction proteins and an increase in the expression of gut IL-17a and IL-2 (Supplementary Figure S6K-P). Further delving into gut microbiota, species abundance, in descending order, was observed as: Young_sur + yFMT, Young_sur + aFMT, Aged_sur + yFMT, and Aged_sur + aFMT (Supplementary Figure S7A-B). The clustering analysis of independent samples indicated distinct microbiota compositions across groups. Nearly all samples within the same group clustered together. Significantly, species such as *Lactobacillus, Allobaculum, Bifidobacterium*, and *Bacteroides* were found at the highest levels with considerable inter-group variations (Supplementary Figure S7C). Changes in α-diversity mirrored the trends seen in microbial abundance. The NMDS analysis highlighted that the β-diversity of the gut microbiota in both young and aged postoperative mice was modulated by FMT (Supplementary Figure S7D-E). Through the combined insights from random forest analysis (Top 10 in genus) and LEfSe, it was ascertained that bacterial genera including *Sutterella, Bifidobacterium, Gemella, Allobaculum, Lactococcus, Akkermansia, Streptococcus, Lactobacillus, Clostridium,* and *Turicibacter* were the most influential in shaping the gut microbial composition (Supplementary Figure S7F-G). Taken together, these findings reinforce the protective role of FMT in mitigating PND in aged mice by modulating the gut microbiota and, consequently, restoring barrier functionality. Nonetheless, the underlying mechanisms warrant further exploration.

**Fig. 8:**
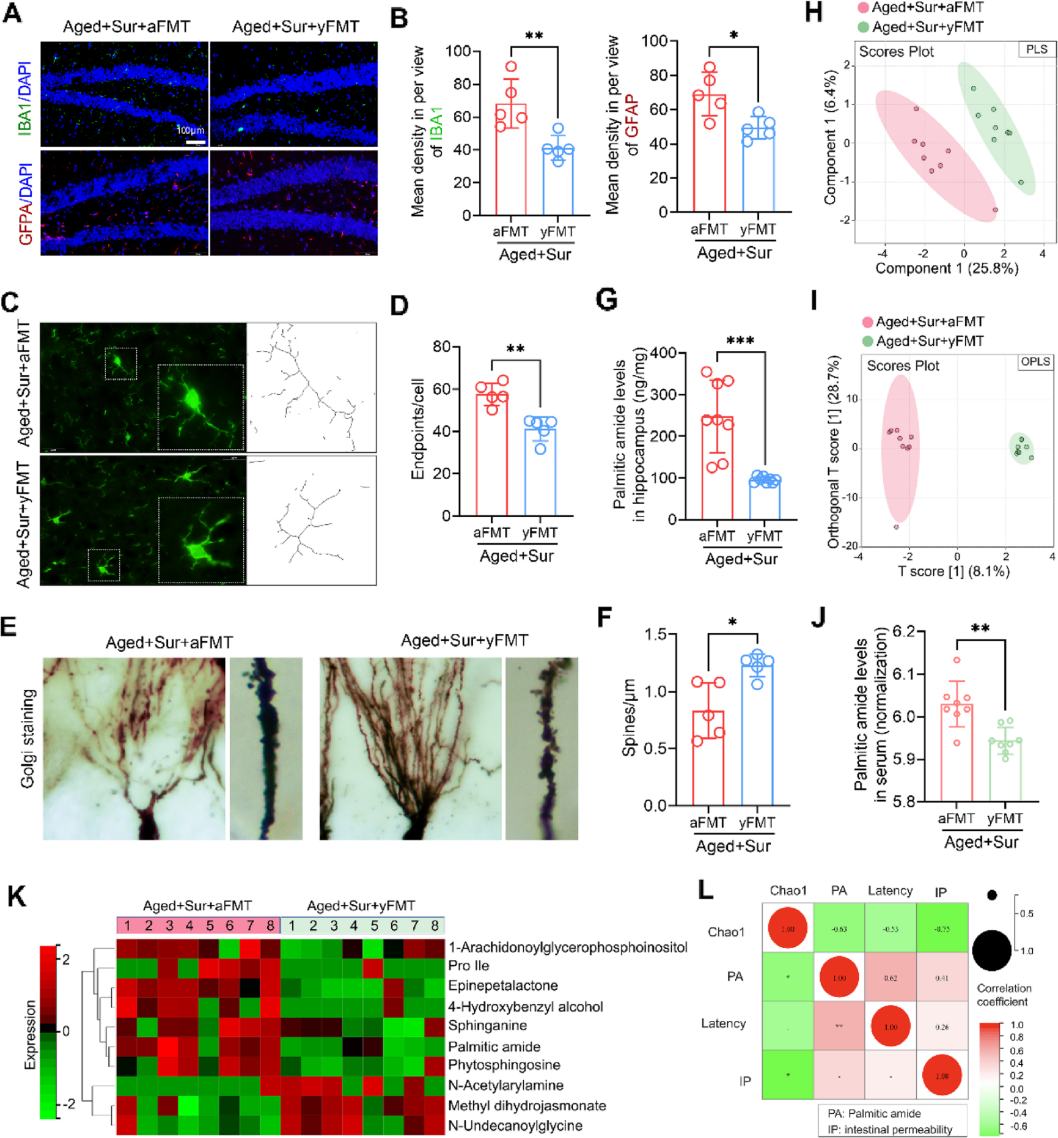
FMT reverses surgery-associated metabolic perturbations and decreases PA contents in old surgery mice. **A** Immunofluorescence staining was used to detect the expression level of the microglial marker IBA1 (green) and astrocyte marker GFAP (red) in the brain hippocampus of aged FMT mice. **B** Correlation with **A**, the fluorescence intensity of IBA1 and GFAP in the brain hippocampus of aged FMT mice was calculated. **C** Confocal laser microscopy magnified the microglia positively expressing IBA1 in the brain hippocampus of aged FMT mice. **D** Correlation with **C**, the number of branches of microglia expressing IBA1 positively in the brain hippocampus of aged FMT mice was calculated. **E** Golgi staining was used to detect morphological changes in neurons and dendrites in the brain hippocampus of aged FMT mice. **F** Correlation with **E**, the length of the spines in each group of mouse neurons under the same magnification. **G** UHPLC analysis was used to detect the PA level in the brain hippocampus of aged FMT mice. **H** Metabolite profiles in the blood of aged FMT mice were analyzed by non-target metabolomics. Principal component analysis of all metabolites detected in the blood of aged FMT mice by orthogonal partial least squares (PLS). **I** Principal component analysis of all metabolites detected in the blood of aged FMT mice by partial least squares (OPLS). **J** Palmitic amide peak areas in blood samples from the two groups of mice were extracted from the metabolic profiles and normalized, and logarithmic values were used to analyze the differences between the two groups. **K** The peak areas of the differential metabolites (10 kinds) in the blood of the two groups of mice were extracted from the metabolic profiles, and a metabolite correlation heatmap was drawn, showing the relative changes in the differential metabolites in each sample. **L** Correlation analysis heatmap of Alpha diversity index (Chao1), PA levels in blood, latency in MWM test and intestinal permeability (area of FITC-dextran) of aged FMT mice. Red represents a positive correlation, green represents a negative correlation, correlation coefficients are indicated in the heatmap. *P < 0.05, **P < 0.01, ***P < 0.001, ****P < 0.0001

To clarify the direct relationship between surgery-induced changes in gut microbiota, barrier functionality, and circulating metabolites, the serum non-target metabolic profile of FMT mice was examined, in addition to evaluating the altered brain histopathology in FMT mice. The results of immunofluorescence staining showed that compared with FMT from aged mice, FMT from young mice significantly reduced the activation of microglia and astrocytes in the hippocampus of brain tissue, which was manifested as the number of IBA1 and GFAP fluorescence-positive cells and a decrease in intensity (Fig. 8A and B). Compared with FMT from aged mice, FMT from young mice inhibited the appearance of activated microglia, with more branches and reduced cell bodies in microglia (Fig. 8C and D). Golgi staining revealed more abundant ganglia in the dendrites and axons of the brain tissue neurons of the FMT from young mice when compared with the FMT from aged mice (Fig. 8E and F). These results suggest that normalization of the gut microbiota in aged mice prevents surgically induced activation of microglia and astrocytes and neuronal damage in the brain tissue of aged mice.

The results of UHPLC analysis showed that compared with FMT from aged mice, FMT from young mice decreased the levels of PA in the hippocampus of aged surgical mice (Fig. 8G). Anofinic acid could not be detected in the brains of aged surgical mice with FMT due to being below the detection limit of it in brain (results not shown). The load and principal components were extracted from the non-target metabolic spectrum of the blood, PLS and OPLS showed that the metabolite profiles of the two groups of mice were significantly different (Fig. 8H and I). In addition, among the 85 kinds detected and determined non-target metabolites in the serum of the FMT mice (available in “figshare”, in “Availability of data and materials” section), 10 kinds of metabolites showed significant changed between the aged surgical mice with FMT from aged mice and with FMT from young mice. Among the ten significant changed metabolites, the level of PA decreased significantly by FMT from young mice (Fig. 8J and K). The results of the correlation analysis of Alpha diversity index (Chao1), PA levels in blood, latency in MWM test, and intestinal permeability (area of FITC-dextran) of aged FMT mice showed that the diversity of gut microbiota was significantly negatively correlated with serum PA levels, latency, and intestinal permeability (Fig. 8L). This indicates that increased PA due to surgery-related gut microbiota abnormalities may be responsible for the development of PND, and regulation of gut microbiota could improve PND by reducing circulating and intracerebral PA content.

### Oral PA induces postoperative cognitive impairment and brain pathological changes in young mice

We focused on the PA, which was found to be the most difference in the metabolite analysis. Following oral administration of 50 mg/kg PA to young (2-month-old) mice, a time-dependent increase in PA levels was observed. UHPLC analysis validated the heightened PA concentrations in the serum (Supplementary Figure S8A) and crucially, within the brain’s hippocampus (Supplementary Figure S8B), suggesting that PA can effectively permeate the blood–brain barrier and amass in brain tissues. Coinciding with the surge in PA levels in the brain was a conspicuous indication of neuroinflammation. Specifically, enhanced immunofluorescence staining showcased a significant augmentation of IBA1 (Supplementary Figure S8C and D) and GFAP (Supplementary Figure S8C and E) in the brain tissues, denoting activated microglia and astrocytes, respectively. However, the staining intensity of NeuN remained unchanged (Supplementary Figure S8C and F). These findings collectively emphasize PA’s capability to breach the blood–brain barrier, accumulate in the brain, and subsequently trigger neuroinflammation.

**Fig. 9:**
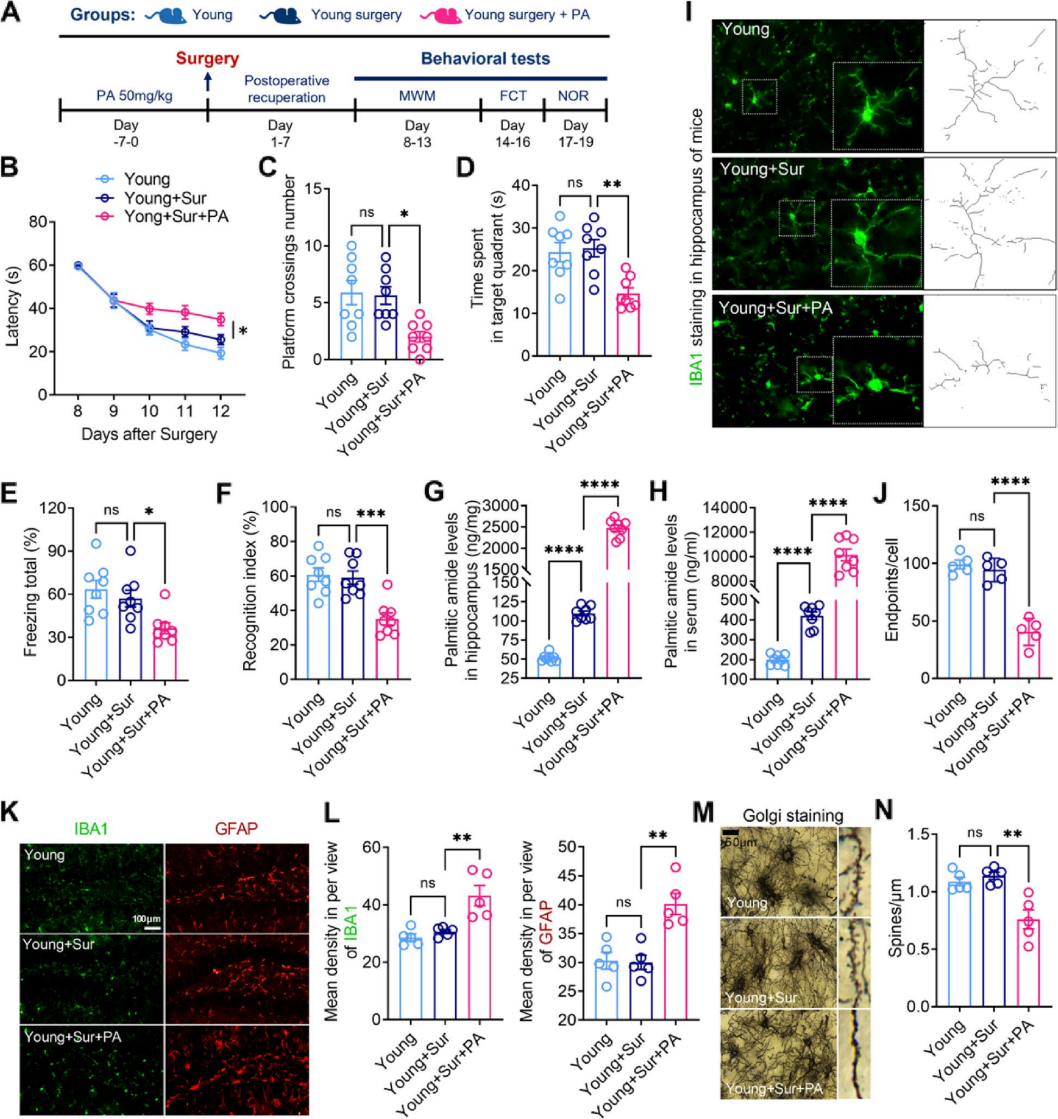
Oral PA induces postoperative cognitive impairment and brain pathological changes in young mice. **A** Flow chart of the experiment indicates the pretreatment of PA in young before surgery and the detection time of each cognitive behavior. **B** Latency to find the platform in the MWM test from 8 to 12 days after surgery of the young mice. **C** The number of times that the young mice crossed the platform in the MWM test in the 13th day after surgery. **D** The retention time in the platform quadrant in the MWM test in the 13th day after surgery of the young mice. **E** Freezing time in the fear conditioning test in the 16th day after surgery of the young mice. **F** Novel object exploration index in novel object recognition in the 19th day after surgery of the young mice. **G** UHPLC analysis was used to detect the PA level in the brain hippocampus of young mice. **H** UHPLC analysis was used to detect the PA level in the serum of young mice. **I** Confocal laser microscopy magnified the microglia positively expressing IBA1 in the hippocampus of mouse brain tissues in each group. **J** Correlation with **I**, the number of branches of microglia expressing IBA1 positively in the hippocampus of the mouse brain tissues of each group was calculated. **K** Immunofluorescence staining was used to detect the expression level of the microglial marker IBA1 (green) and astrocyte marker GFAP (red) in the hippocampus of mouse brain tissues in each group. **L** Correlation with **K**, the fluorescence intensity of IBA1 and GFAP in the hippocampus of the mouse brain tissues in each group was calculated. **M** Golgi staining was used to detect morphological changes in neurons and dendrites in the hippocampus of mice in each group. **N** Correlation with **M**, the length of the spines in each group of mouse neurons under the same magnification. *P < 0.05, **P < 0.01, ***P < 0.001, ****P < 0.0001, ns means no significant

To directly test the effect of PA on PND, young mice were orally supplemented daily with 50 mg/kg PA for 7 days. The experimental flowchart indicates the time points at which the young mice were manipulated (Fig. 9A). The results of the MWM showed that young mice treated with PA had significantly increased postoperative escape latency compared to the young surgical mice without PA treatment (Fig. 9B). PA significantly decreased the platform crossing time and the time spent in the platform quadrant of young surgical mice (Fig. 9C and D). In the FCT, PA significantly decreased the postoperative freezing time of the young surgical mice (Fig. 9E). Similarly, in the NORT, the mice treated with PA spent less time exploring new objects after surgery and showed a higher recognition index, indicating memories of familiar objects (Fig. 9F). These results suggest that PA exacerbated surgery-induced cognitive impairment in young mice.

The levels of PA in the blood and hippocampus of different treated young mice were subsequently measured using UHPLC. The results showed that the surgical factor alone in young mice resulted in an approximately twofold increase in PA content in the circulation and brain. However, 50 mg/kg gavage for seven consecutive days resulted in an approximately 20-fold increase in PA levels in the blood and brain of young surgical mice (Fig. 9G and H). PA administration in young surgical mice caused the microglia in the hippocampus transform from a complex morphology with small nuclei and long protrusions to an activated morphology, which turned into enlarged and thickened cell bodies with reduced branching, these changes were not observed in the hippocampus of young mice underwent surgery without PA treatment (Fig. 9I and J). The results of immunofluorescence staining showed that compared with young surgical mice, PA administration in young surgical mice significantly increased the activation of microglia and astrocytes in the hippocampus of brain tissue, which was manifested as the number of IBA1 and GFAP fluorescence-positive cells and an increase in intensity (Fig. 9K and L). Golgi staining showed that the number of ganglia in the dendrites and axons of the young surgical mice decreased with the PA treatment, while surgical factors alone had no significant effect on the neurons of the young mice (Fig. 9M and N). These results confirm that surgery-induced dysbiosis of gut microbiota and its associated increase in PA levels play a key role in promoting PND.

## Discussion

PND increases the risk of future brain dysfunction, including dementia, Alzheimer’s disease, and long-term cognitive decline, making it a symptom of clinical interest [21]. As the world population ages, increasing numbers of older adults will inevitably undergo surgery, elderly patients are characterized by a high incidence of PND [22]. Here, we demonstrate that surgery causes abnormalities in the gut microbiota and surgery-driven gut microbial dysbiosis contribute to cognitive dysfunction in at least two ways. On the one hand, surgery-driven gut microbial dysbiosis increase intestinal permeability, and on the other hand, it promotes cognitive impairment by altering the composition of metabolites, especially by increasing PA production.

In the present study, we first compared cognitive function, activation of microglia and astrocytes, and changes in neuronal dendritic spines in young and old mice with or without surgery, and examined alterations in gut microbiota. We found that in all three behavioral tests, surgery resulted in significant cognitive impairment in aged mice, whereas young mice did not show reduced cognitive function. Activation of microglia following peripheral surgery has been described in human subjects and various rodent surgical models and is associated with persistent cognitive impairment [23, 24]. Astrocyte activation is also considered to be an important factor in neuroinflammation [25]. To date, most studies focusing on PND in rodent models have focused on the hippocampus, given its important role in learning and memory [26]. In the current study, we labeled microglia and astrocytes in the mouse hippocampus using IBA1 and GFAP and observed the number of dendritic spines using Golgi staining. The changes in these brain cells were consistent with previous reports, but it is important to emphasize that in the model we used (exploratory laparotomy), the activation of microglia and astrocytes, as well as dendritic spine loss, was only observed in aged mice after surgery.

However, both young and old mice showed dysbiosis of gut microbiota after surgery. Some hallmark gut microbiota species changed considerably after surgery, including a significant increase in *Bacteroides* and a significant decrease in *Akkermansia*. At present, only a few basic studies focus on gut microbiota and perioperative cognition [27–29], in the current study, we complement this evidence and provide a profile of changes in the gut microbiota of young and old mice with or without surgery. Surgery in both young and old mice induced a downregulation of diversity and abundance of gut microbiota, and in particular it led to alterations in some specific flora, such as *Akkermansia* that mentioned above, which has been reported to have beneficial effects in cognitive function [30]. It was of interest that why the surgery led to changes in the gut microbiota in both young and aged mice, but it just caused cognitive dysfunction and neuropathological changes in aged mice. The exact mechanism through which laparotomy exerts this effect remains to be fully elucidated.

The mechanisms through which laparotomy affects gut microbiota remain intricate and multifaceted. Pain, which is intrinsically associated with surgical interventions such as laparotomy, seems to play a paramount role in modulating the microbial milieu of the gut [31]. Conditions like IBS, characterized by abdominal discomfort, have demonstrated the ability to affect key gastrointestinal functions, which subsequently alter the gut microbial profile [32]. Given the inevitable pain post-laparotomy, it stands to reason that such surgical interventions might induce significant shifts in the gut microbial dynamics [33]. Moreover, the bidirectional links between the gut and the brain, predominantly mediated by neurotransmitters, hold potential keys to unraveling the susceptibility of aged mice to PND following laparotomy. The synergy between age-related intestinal barrier dysfunction [34], and surgery-induced pain may intensify neuroinflammatory responses in PND models.

We then focused our attention on intestinal barrier function and permeability, as intestinal tract is the tissue in direct contact with the microbiota and has been reported to function in an age-related manner [34, 35]. We evaluated the intestinal permeability, blood flow, and tight junction protein expression in mice. As we speculated, naïve aged mice had significantly increased intestinal permeability and blood flow and significantly decreased tight junction protein expression compared to naïve young mice. These physiological status changes of intestinal tract of aged mice may be resulted in the aged mice being more sensitive to surgery and the surgery-driven gut microbial dysbiosis. The current results show that intestinal permeability significantly increased in aged surgery, whereas in young mice, surgery had no significant effect on intestinal permeability, blood flow, and tight junction protein expression. Although surgery led to changes in gut microbiota in mice, it did not affect intestinal function in young mice, which may explain the normal cognitive function of young mice in the postoperative period. To clarify whether there is a direct relationship between intestinal permeability increase and the gut microbial dysbiosis in aged mice in the postoperative period, we performed FMT on aged surgical mice, which received feces from healthy young mice and aged mice, respectively. Our results indicate that correcting the disorganized gut microbiota in aged surgical mice via FMT can improve cognitive function by reducing inflammation and neuropathological changes in the brain, this further supports the idea of a functional link between the gut microbiota, neuroinflammation, and brain function [36]. Moreover, compared with FMT from aged mice, FMT from young mice reduced intestinal permeability and increased colonic tight junction protein expression in aged surgical mice, demonstrating a direct effect of gut microbiota on barrier function in aged mice in the postoperative period.

In the current study, various interventions were employed, mainly including age, surgery, FMT, anti-inflammatory agents, probiotic supplementation, and metabolite supplementation. These different treatments facilitated the identification of specific bacterial species modulated by various factors such as age, surgery, anti-inflammation, and fecal microbiota transplantation, as well as the impact of these specific bacteria on PND. Our primary focus was on the surgical factor, which significantly affected the abundance of various bacteria represented by *Akkermansia* and *Bacteroidetes*. Two independent FMT experiments screened some of the most abundant species or those with the most influential impact on the gut microbiota composition of elderly postoperative mice. The former included *Akkermansia*, *Bacteroidetes*, *Verrucomicrobia*, and *Cyanobacteria*, while the latter included *Sutterella*, *Bifidobacterium*, *Gemella*, *Allobaculum*, *Lactococcus*, *Akkermansia*, *Streptococcus*, *Lactobacillus*, *Clostridium*, and *Turicibacter*.

In fact, some of the bacteria mentioned above have been reported to influence neuroinflammation. For instance, the *Bacteroides fragilis* has been reported to cross biophysical barriers and secrete an unusually complex mixture of neurotoxins, including the highly pro-inflammatory lipopolysaccharide, promoting neuroinflammation [37]. *Verrucomicrobia* has been documented to be significantly increased in PD patients and closely associated with circulating inflammatory factors such as IL-1β, IL-2, IL-4, IL-6, IL-13, IL-18, GM-CSF, IFNγ, and TNF-α. *Lactococcus lactis subsp* has been reported to treat an LPS-induced depression-like model in mice [38]. We consider *Akkermansia* to be a species of interest, as our findings highlighted that the postoperative decrease in *Akkermansia* was significantly increased by FMT from young mice, and both clinical and animal studies have shown that *Akkermansia* supplementation can improve metabolic diseases [39, 40].

To further clarify the role of intestinal barrier function in the development of PND, we gave a barrier-enhancing substance to aged mice and observed whether it could prevent the development of cognitive disorder in aged mice in the postoperative period. Recently, we demonstrated that IPA has a direct function in enhancing intestinal barrier in obese mice [14], so we tried to use it on aged surgical mice. As we expected, IPA prevented the development of PND in aged mice and increased the expression of intestinal tight junctions in aged surgical mice. Deficiently, although we demonstrated that IPA promoted the intestinal barrier in aged surgical mice, it could not be ruled out whether IPA exerted its effect directly by entering the brain tissue of mice, for studies have shown that many short-chain fatty acids can directly act on the central nervous system to reduce neuroinflammation and restore cognitive function [41, 42]. An increase in intestinal inflammation is one of the reasons for barrier function impairment. We observed an elevation in the expression of IL-17a and IL-22 in the ileum of aged mice, as well as the augmentative effects of surgery on them and the suppressive role of the anti-inflammatory agent, dexamethasone. Given the suppressive effects of treatments like IPA1 and FMT on IL-17a and IL-22, this suggests that the gut microbiota or gut metabolites may influence intestinal inflammation and barrier function, potentially playing a role in the development of PND.

As a large number of studies report the effect of different bacterial metabolites on intestinal barrier function and cognitive function [41–44], and IPA has been proven to be important metabolites of the gut microbes [45], we speculate that surgery-driven gut microbial dysbiosis may influence the development of PND by altering certain other metabolites. Although the study has shown that probiotic supplementation and gut microbiota metabolites such as short-chain fatty acids can alleviate PND [46], studies that directly focus on and demonstrate the link between gut microbiota and metabolites observed in PND are few and far between. We delineated the overall non-target metabolite profiles in young and old mice with or without surgery. These screened differential metabolites, which are described in detail in the “Results” section, may be the main reason why the gut microbiota drives central lesions through the periphery. Some hallmark metabolites showed significant change after surgery, such as a significant decrease in anofinic acid and a significant increase in palmitic amide.

To demonstrate whether these metabolite changes were caused by surgery-driven gut microbial dysbiosis, we performed non-target metabolomics again in aged mice with FMT, and we found that FMT from young mice significantly altered the metabolic profile of aged surgical mice. Harnessing endogenous pathways and mediators offers a unique opportunity to reduce inflammation after surgery, without causing unnecessary side effects [47]. We expected to find some beneficial metabolites, but the results showed that only 10 kinds of metabolites changed in the blood of aged surgical mice after FMT from young mice. Typically, anofinic acid was reduced extremely in aged mice, but FMT failed to increase its levels in aged surgical mice, which may suggest that anofinic acid is not relevant to gut microbiota. Although we were unable to identify beneficial metabolites that related to gut microbiota, we identified representative metabolites that promote the development of PND, such as palmitic amide (HMDB12273, PA). Compared to young mice, PA was significantly increased in aged mice, and their levels were further increased both in young and aged mice when surgery was performed. More importantly, PA was significantly reduced in aged surgical mice when they received FMT from young mice. PA is a primary fatty acid amide coming from palmitic acid (C16:0) [48]. Key questions remain regarding how these lipid amides are produced and degraded in biological systems. We acknowledge that there is limited study discussing the function of palmitic amide, especially its influence on intestinal barrier integrity and PND.

Finally, to verify whether PA is a key metabolite in promoting PND, we gave continuous oral administration of PA in young mice and observed the effect of surgery on them. Earlier, we thought that giving PA to young mice might not affect their cognition function under the surgery condition. We thought that young mice possess a good intestinal barrier function, which may prevent PA from entering the bloodstream. However, after performing none-target metabolomics and assaying PA levels in brain tissue of young and young surgery mice, we found that PA levels in blood and brain were significantly increased despite the good intestinal barrier function in the young surgery mice. This suggests that the increase in PA levels in blood and brain is associated with normal absorption function of intestinal tract, independent of the intestinal permeability. As the evidence shows, the administration of PA gavage to young mice resulted in at least a 20-fold increase in intracerebral and circulating levels of PA, whereas surgery resulted in only an approximately onefold increase in PA levels in mice. In the presence of high circulating PA levels, surgery led to cognitive impairment and brain pathological changes in young mice. This result not only confirms that PA enters the bloodstream through absorption function of intestinal tract, but also confirms that it is a key gut microbiota metabolite that promotes the development of PND.

In conclusion, our findings suggest that in the context of surgery, the development of PND is affected by age; in particular, changes in gut microbial composition due to aging are one of the key factors. Our results highlight surgery-driven gut microbial dysbiosis and that such abnormalities contribute to cognitive dysfunction in at least two ways. First, it increases intestinal permeability, and second, it induces abnormal microbiota-related metabolite changes. More importantly, our findings may have implications in the diagnosis and treatment of PND. It provides therapeutic strategies for PND by remodeling of the gut microbiota and by supplementing with beneficial microbiota or their metabolites. Combinations of specific bacteria (e.g., *Akkermansia and Lactobacillus*) and metabolites (e.g., palmitic amide) may serve as biomarkers for the early diagnosis of PND and this deserves further validation in a large cohort of PND patients.

### Supplementary Information

**Additional file 1: Figure S1.** Effect of exploratory laparotomy on body weight and food intake in young and aged mice over a 14-day post-surgery period. **Figure S2.** Comparative composition of gut microbiota in young and aged mice subjected to surgery and with or without behavioral tests. **Figure S3.** Influence of antibiotics vancomycin and ampicillin on gut microbiota diversity in mice prior to surgery. **Figure S4.** Impacts of dexamethasone on cognition and intestinal function in young and aged surgical mice. **Figure S5.** Impacts of dexamethasone on gut microbiota in aged surgical mice. **Figure S6.** Impacts of FMT on cognition and intestinal function in young and aged surgical mice. **Figure S7.** Impacts of FMT on gut microbiota in young and aged surgical mice. **Figure S8.** PA oral administration increases the brain PA level in young mice and leads to neuroinflammation.

## Data Availability

16S rRNA gene sequencing raw data that support the findings of this study are available in https://www.ncbi.nlm.nih.gov/. The BioProject ID in Sequence Read Archive (SRA) of NCBI is PRJNA923318. The submission name is: Surgery-induced gut microbial dysbiosis promotes cognitive impairment in aged mice. The metabolomics data that support the findings of this study are available in “figshare” at http://doi.org/10.6084/m9.figshare.19556161.

## Abbreviations

Abx: Antibiotics
CNS: Central nervous system
FCT: Fear conditioning test
FMT: Fecal microbiota transplant
GI: Gastrointestinal
IPA: Indole propionic acid
MWM: Morris water maze
NORT: New object recognition test
PA: Palmitic amide
DEX: Dexamethasone
*Lac.*: Lactobacillus acidophilus
PND: Perioperative neurocognitive disorders
