# Supplementary material for: Surgery-induced gut microbial dysbiosis promotes cognitive impairment via regulation of intestinal function and the metabolite palmitic amide
Source: Microbiome. 2023 Nov 8;11:248. doi: 10.1186/s40168-023-01689-6 (PMC10631187; doi:10.1186/s40168-023-01689-6)
Supplement: Supplementary file 2 — Additional file 1: Figure S1. Effect of exploratory laparotomy on body weight and food intake in young and aged mice over a 14-day post-surgery period. Figure S2. Comparative composition of gut microbiota in young and aged mice subjected to surgery and with or without behavioral tests. Figure S3. Influence of antibiotics vancomycin and ampicillin on gut microbiota diversity in mice prior to surgery. Figure S4. Impacts of dexamethasone on cognition and intestinal function in young and aged surgical mice. Figure S5. Impacts of dexamethasone on gut microbiota in aged surgical mice. Figure S6. Impacts of FMT on cognition and intestinal function in young and aged surgical mice. Figure S7. Impacts of FMT on gut microbiota in young and aged surgical mice. Figure S8. PA oral administration increases the brain PA level in young mice and leads to neuroinflammation. [file 40168_2023_1689_MOESM1_ESM.docx]

**Surgery-induced gut** **microbial dysbiosis promotes cognitive impairment via** **regulation of intestinal function and the metabolite palmitic amide**

Cailong Pan ^a,1^, Huiwen Zhang ^a,1^, Lingyuan Zhang ^a^, Lu Chen ^a^, Lu Xu ^a^, Ning Xu ^a^, Xue Liu ^a^, Qinghai Meng ^b^, Xiaoliang Wang ^c,^ *, Zhi-Yuan Zhang ^a, d,^ *

^a^ School of Basic Medical Sciences, Nanjing Medical University, Nanjing 211166, China

^b^ School of Medicine & Holistic Integrative Medicine, Nanjing University of Chinese Medicine, Nanjing 210023, China

^c^ Department of Anesthesiology, Nanjing First Hospital, Nanjing Medical University, Nanjing 210029, China

^d^ Key Laboratory of Rare Metabolic Diseases, Nanjing Medical University, Nanjing 211166, China

^1^ Co-first author

*Address correspondence to: Zhi-Yuan Zhang, Nanjing Medical University, Longmian Avenue 101, Nanjing 211166, China. Email: [zzy@njmu.edu.cn](mailto:zzy@njmu.edu.cn). Tel: +86-25-86869333; Fax: +86-25-86869333. Xiaoliang Wang, Nanjing First Hospital, Nanjing Medical University, Changle Road 89, Nanjing 210029, China. Email: [wxl145381@njmu.edu.cn](mailto:wxl145381@njmu.edu.cn).

**Supplementary Figures**

**
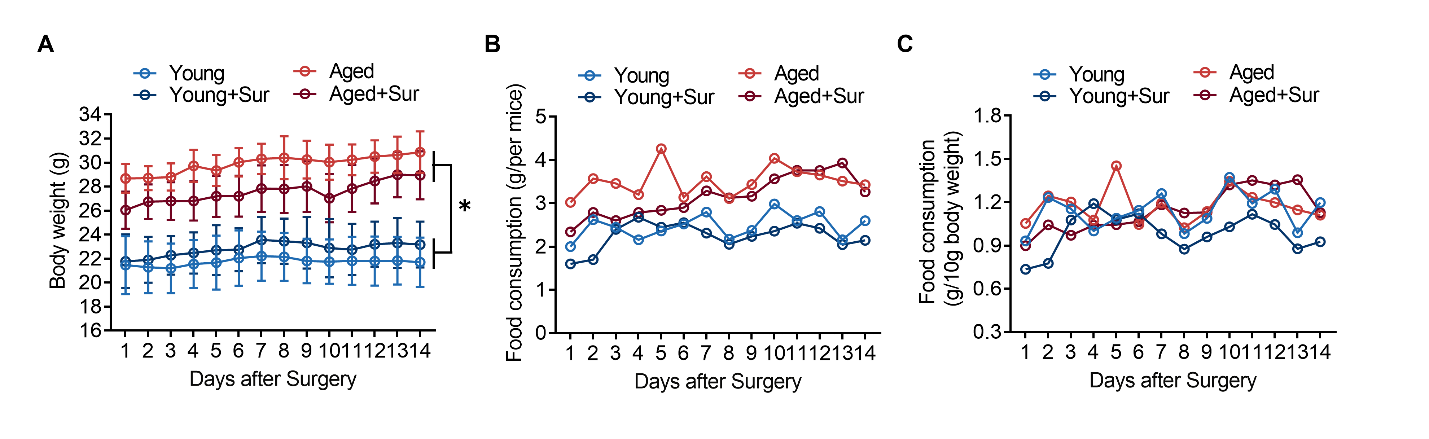
**

**Figure S1. Effect of exploratory laparotomy on body weight and food intake in young and aged mice over a 14-day post-surgery period.**

(A) Changes in body weight of young (2-month-old) and aged (18-month-old) mice subjected to exploratory laparotomy over 14 days, with non-operated young and aged mice serving as controls. (B) Daily food intake for each mouse over the 14-day post-surgery period. (C) Daily food intake normalized to body weight for each mouse over the 14-day post-surgery period.


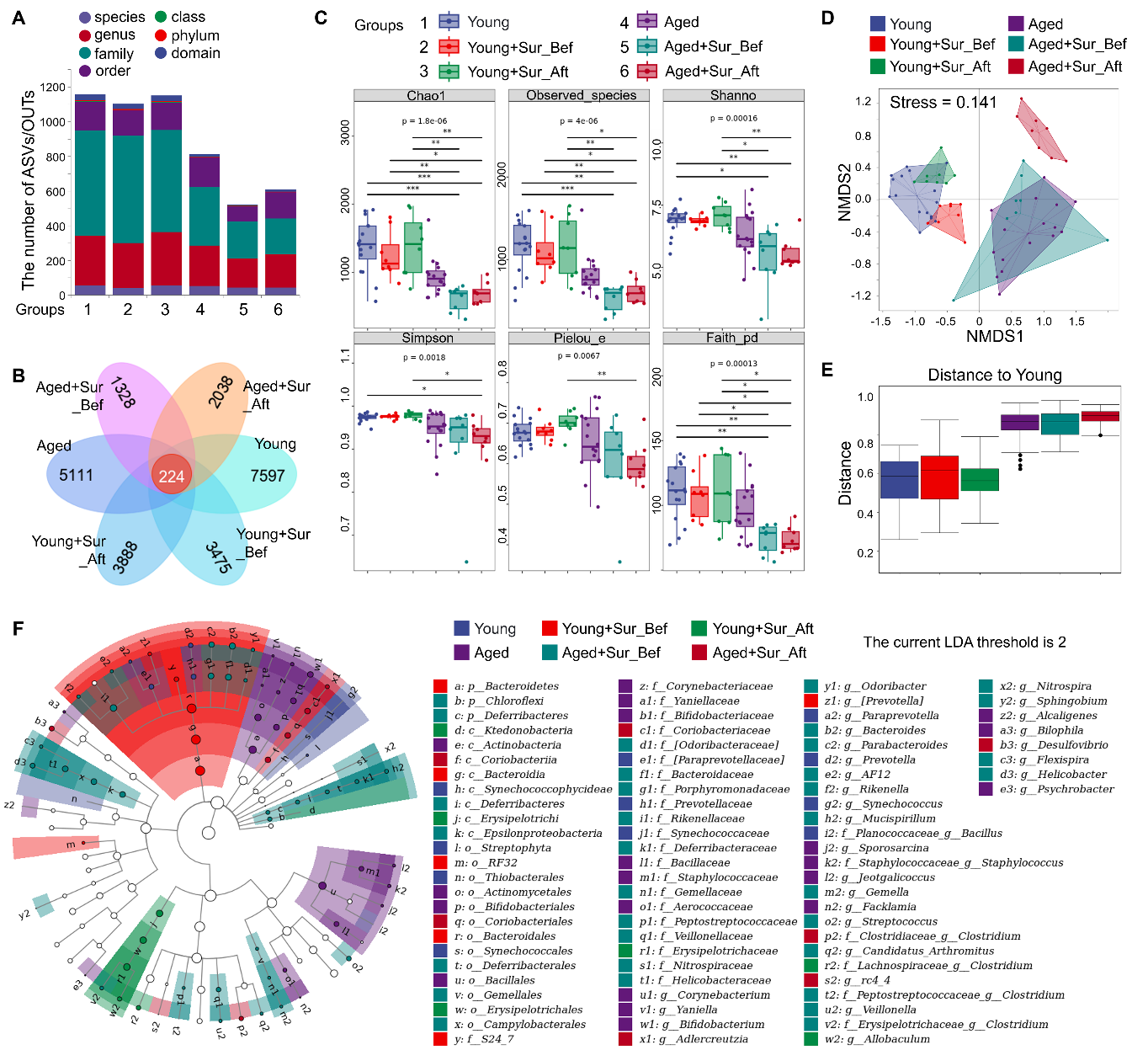


**Figure S2. Comparative composition of gut microbiota in young and aged mice subjected to surgery and with or without behavioral tests**

(A) Bar graph demonstrates species composition variations in the fecal samples, spanning various taxonomic levels. (B) Venn diagram showcases the OTUs identified in fecal samples using 16S rRNA gene sequencing, highlighting shared and unique OTUs across groups. (C) The α-diversity analysis of gut microbiota across all groups. (D) NMDS elucidates the beta diversity of gut microbiota in fecal samples. (E) PERMANOVA analysis assesses the beta diversity within fecal samples. (F) LEfSe analysis pinpoints species with significant variances in fecal samples, using a one-against-all strategy with an LDA threshold of 2.


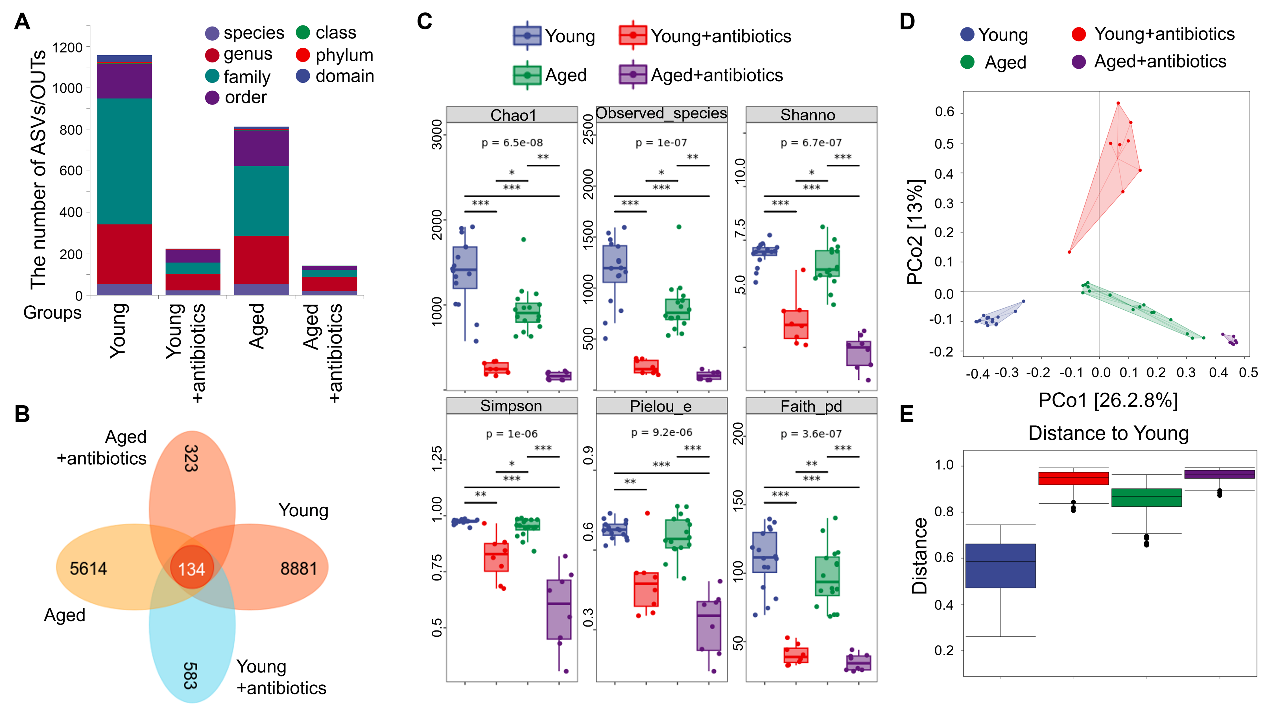


**Figure S3. Influence of antibiotics vancomycin and ampicillin on gut microbiota diversity in mice prior to surgery**

Prior to surgery, mice designated for FMT underwent a seven-day regimen with drinking water infused with antibiotics (vancomycin at 0.5 g/L and ampicillin at 1 g/L) to mitigate their intrinsic gut microbial populations. (A) Bar chart reveals species composition differences across groups. (B) Venn diagram presents OTUs from fecal samples identified via 16S rRNA gene sequencing. (C) Exploration of the α-diversity of fecal-derived gut microbiota. (D) PCoA analysis probes the beta-diversity in mouse fecal samples. (E) PERMANOVA analysis quantifies the beta diversity of gut microbiota.


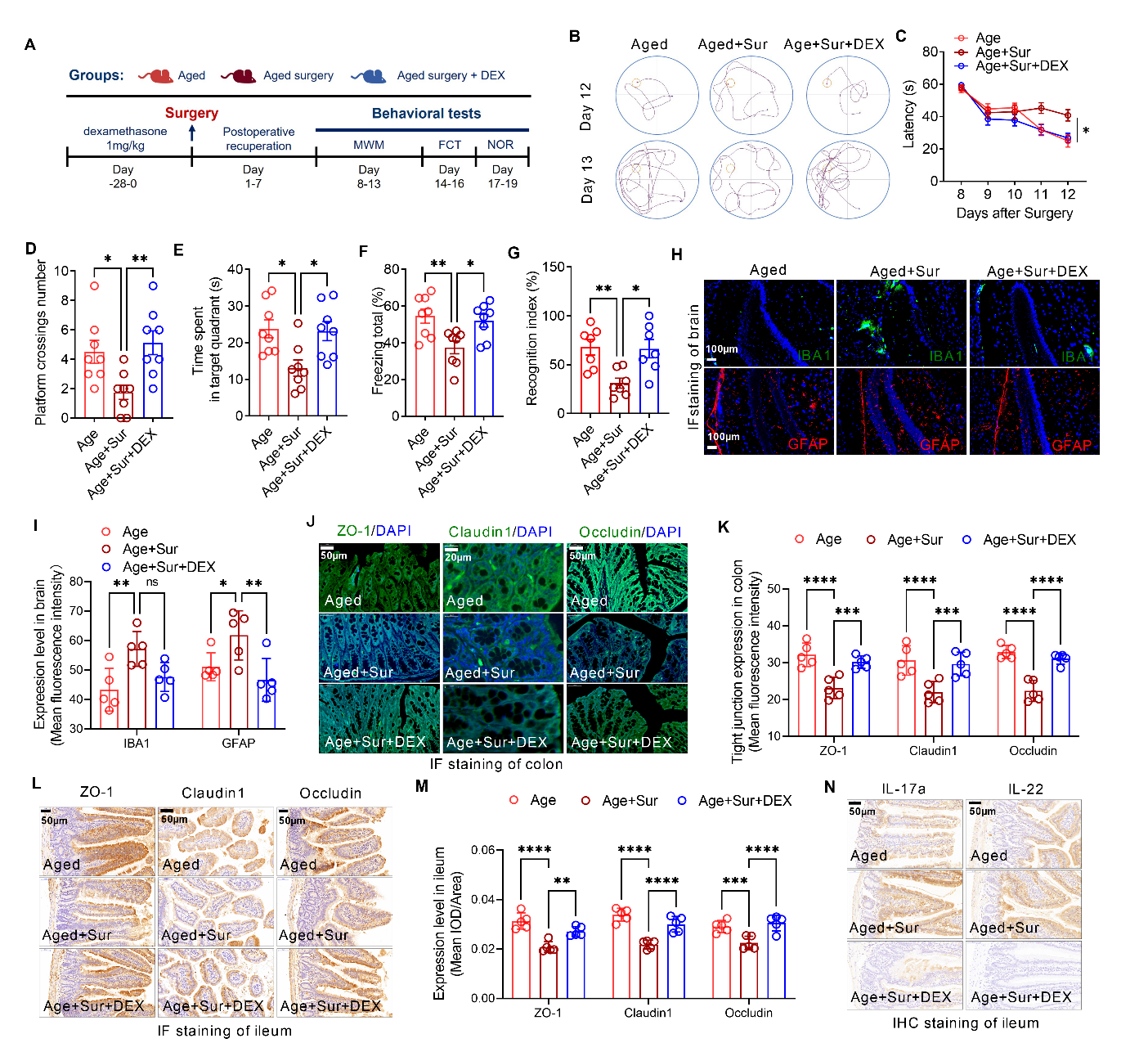


**Figure S4. Impacts of dexamethasone on cognition and intestinal function in young and aged surgical mice**

(A) Flow chart of the experiment indicates the pretreatment of dexamethasone in aged mice before surgery and the detection time of each cognitive behavior. (B) Swimming path diagrams of the aged mice with different treatment in the MWM test on postoperative day 12 and day 13. (C) Latency to find the platform in the MWM test from 8 to 12 days after surgery of the aged mice. (D) The number of times of mice in each group crossed the platform in the MWM test in the 13^th^ days after surgery. (E) The retention time in the platform quadrant in the MWM test in the 13^th^ days after surgery of the aged mice. (F) Freezing time in the fear conditioning test in the 16^th^ days after surgery of the aged mice. (G) Novel object exploration index in novel object recognition in the 19^th^ days after surgery of the aged mice. (H) Representative images of immunofluorescence staining of IBA1and GFAP in brain tissues of the aged mice. (I) Correlation with H, the fluorescence intensity of IBA1 and GFAP in the mouse brain tissues was calculated. (J) Representative images of immunofluorescence staining of the tight junction protein ZO-1, Cluadin-1, and Occludin in colon tissues of the aged mice. (K) Correlation with J, the fluorescence intensity of ZO-1, Cluadin-1, and Occludin in the mouse colon tissues was calculated. (L) Representative images of immunohistochemical staining of the tight junction protein ZO-1, Cluadin-1, and Occludin in ileum tissues of the aged mice. (M) Correlation with L, the mean IOD/area of ZO-1, Cluadin-1, and Occludin in the mouse ileum tissues was calculated. (N) Immunohistochemical staining was used to detects the expression level of IL-17a and IL-22 in ileum tissues of the aged mice. **P <* 0.05, ***P <* 0.01, ****P <* 0.001, *****P <* 0.0001.


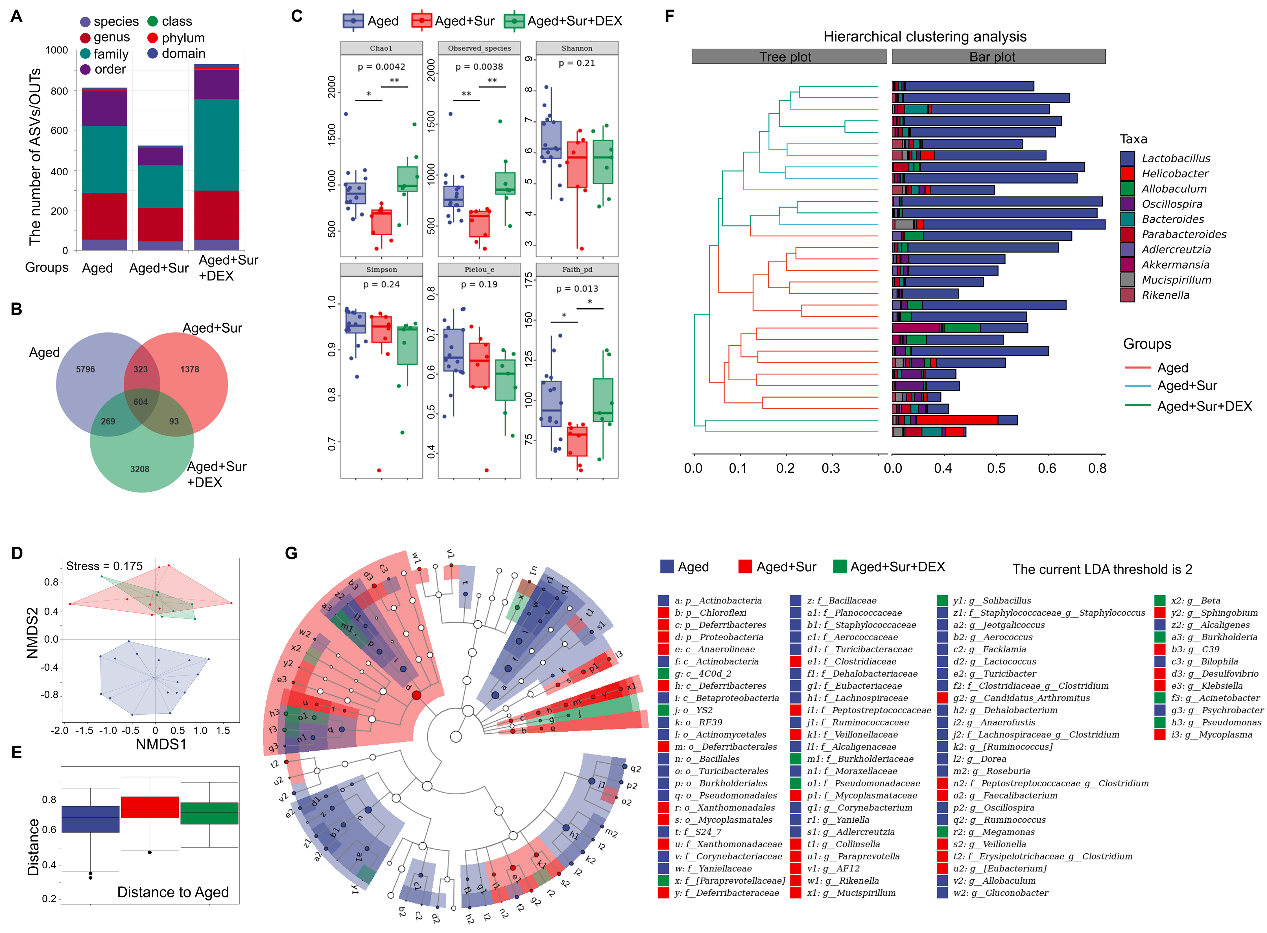


**Figure S5. Impacts of dexamethasone on gut microbiota in aged surgical mice**

(A) Bar chart presents species composition variations in mouse fecal samples. (B) Venn diagram indicates the OTUs identified in fecal samples. (C) Alpha diversity analysis of gut microbiota in fecal samples. (D) NMDS evaluates beta diversity of fecal gut microbiota. (E) PERMANOVA analysis quantifies the beta diversity of gut microbiota. (F) Hierarchical clustering emphasizes fecal sample similarity across groups. (G) LEfSe analysis detects species with pronounced differences in fecal samples.


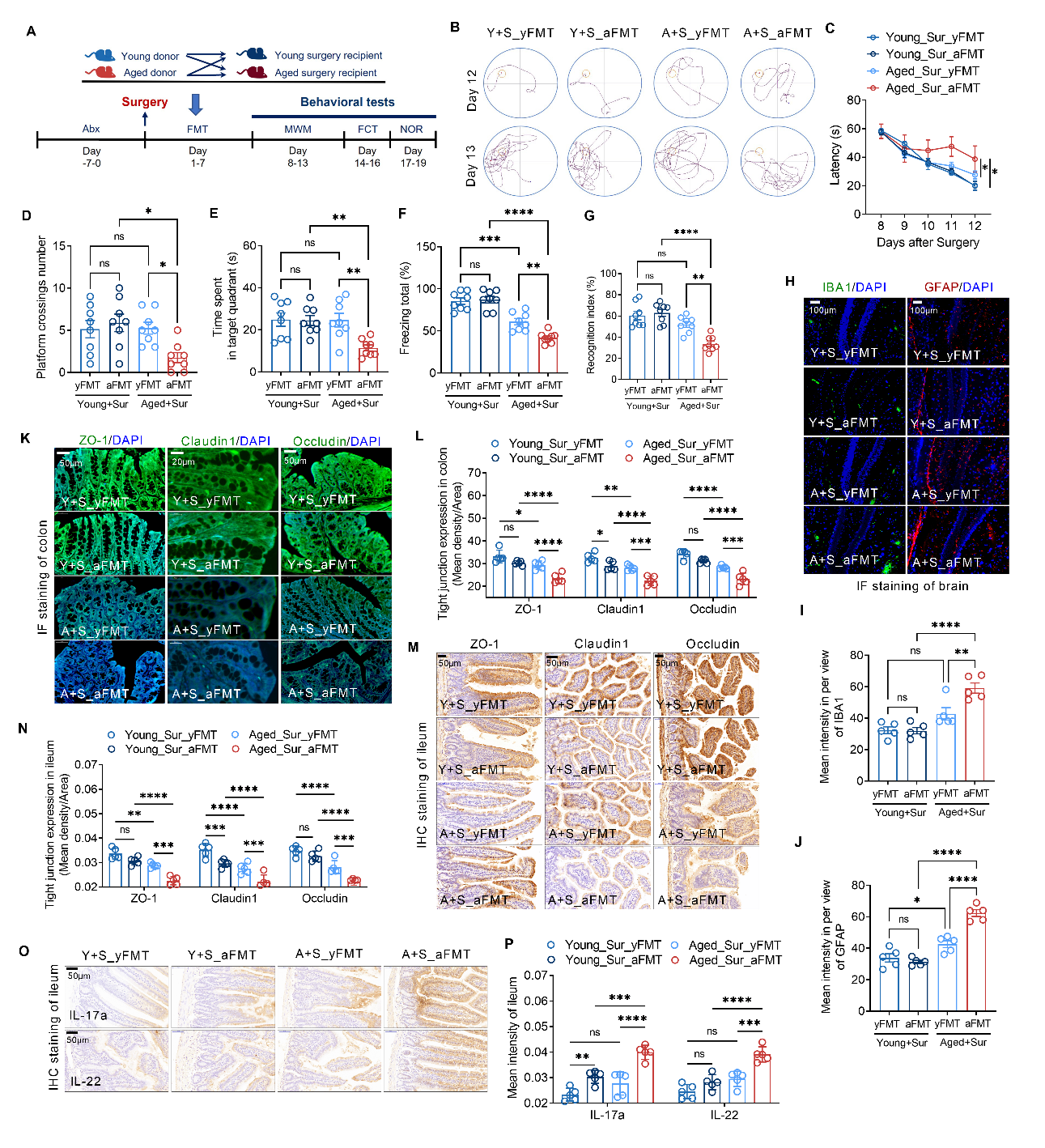


**Figure S6. Impacts of FMT on cognition and intestinal function in young and aged surgical mice**

(A) Flow chart of the experiment indicates the handling of the donor and recipient mice in the FMT experiment and the detection time of each cognitive behavior. (B) Swimming path diagrams of the recipient mice in the MWM test on postoperative day 12 and day 13. (C) Latency to find the platform in the MWM test from 8 to 12 days after surgery of the recipient mice. (D) The retention time of the recipient mice in the platform quadrant in the MWM test in the 13^th^ days after surgery. (E) The number of times that the recipient mice crossed the platform in the MWM test in the 13^th^ days after surgery. (F) Freezing time in the fear conditioning test in the 16^th^ days after surgery of the recipient mice. (G) Novel object exploration index in novel object recognition in the 19^th^ days after surgery in the recipient mice. (H) Representative images of immunofluorescence staining of IBA1and GFAP in brain tissues of the recipient mice. (I) Correlation with H, the fluorescence intensity of IBA1 in the mouse brain tissues was calculated. (J) Correlation with H, the fluorescence intensity of GFAP in the mouse brain tissues was calculated. (K) Representative images of immunofluorescence staining of the tight junction protein ZO-1, Cluadin-1, and Occludin in colon tissues of the recipient mice. (L) Correlation with K, the fluorescence intensity of ZO-1, Cluadin-1, and Occludin in the mouse colon tissues was calculated. (M) Representative images of immunohistochemical staining of the tight junction protein ZO-1, Cluadin-1, and Occludin in ileum tissues of the recipient mice. (N) Correlation with M, the mean IOD/area of ZO-1, Cluadin-1, and Occludin in the mouse ileum tissues was calculated. (O) Immunohistochemical staining was used to detects the expression level of IL-17a and IL-22 in ileum tissues of the recipient mice. (P) Correlation with O, the mean IOD/area of IL-17a and IL-22 in ileum was calculated. **P <* 0.05, ***P <* 0.01, ****P <* 0.001, *****P <* 0.0001.

**
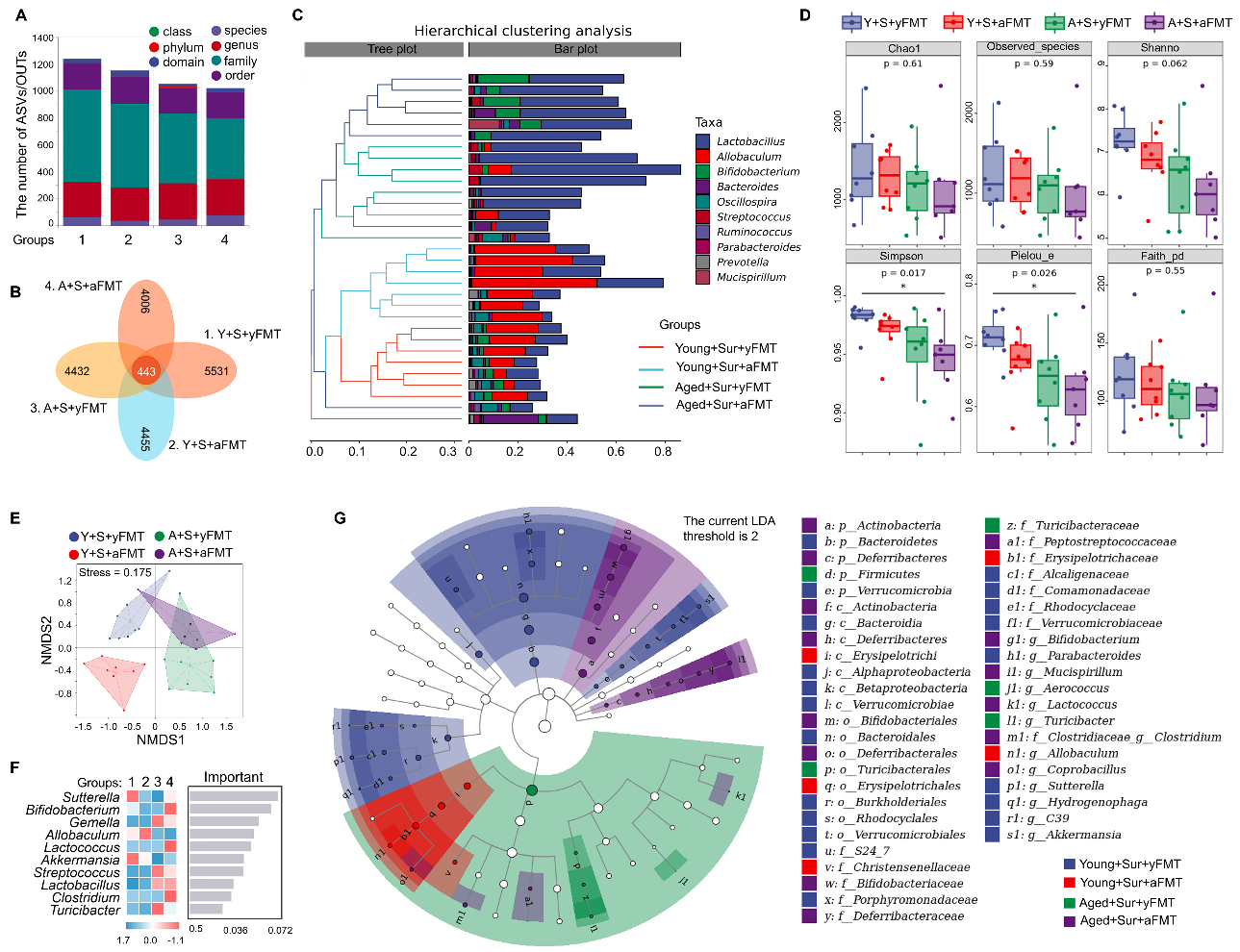
**

**Figure S7. Impacts of FMT on gut microbiota in young and aged surgical mice**

(A) Bar graph showcases species composition variations in mouse fecal samples. (B) Venn diagram delineates OTUs from fecal samples. (C) Hierarchical clustering illustrates fecal sample likeness across groups. (D) Analysis of the α-diversity of gut microbiota. (E) NMDS investigates the beta diversity in fecal samples. (F) Random forest analysis of fecal species composition denotes the influence of specific microbes on compositional variations. (G) LEfSe distinguishes species with significant disparities in fecal samples.

**
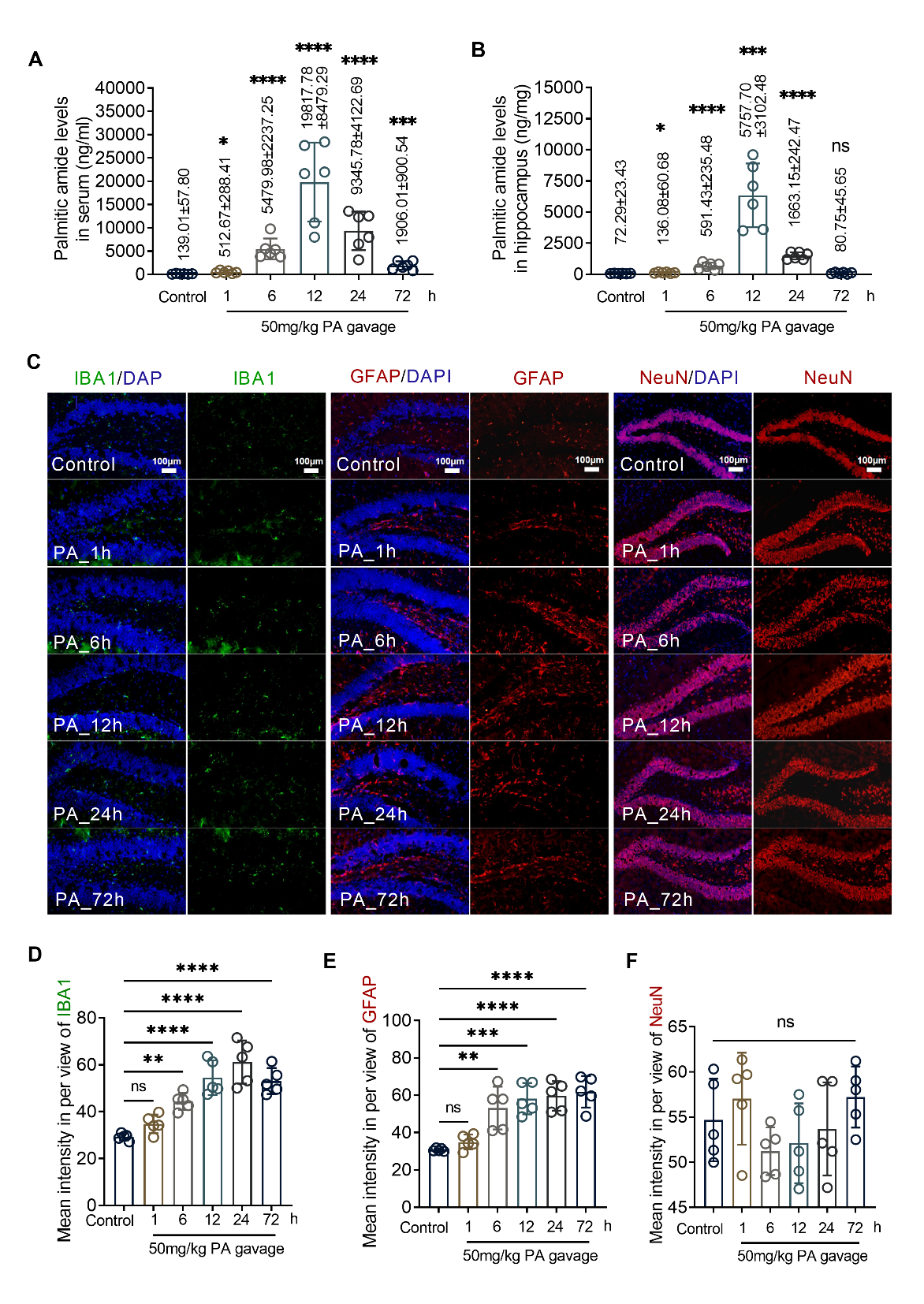
**

**Figure S8. PA oral administration increases the brain PA level in young mice and leads to neuroinflammation**

Young (2-month-old) mice were gastric gavage with 50 mg/kg PA for 1, 6, 12, 24, or 72 h. (A) UHPLC analysis was used to detect the PA level in the serum of mice. (B) UHPLC analysis was used to detect the PA level in the brain hippocampus of mice. (C) Representative images of immunofluorescence staining of IBA1, GFAP and NeuN in brain tissues of mice. (D) Correlation with C, the fluorescence intensity of IBA1 in the mouse brain tissues was calculated. (E) Correlation with C, the fluorescence intensity of GFAP in the mouse brain tissues was calculated. (F) Correlation with C, the fluorescence intensity of NeuN in the mouse brain tissues was calculated. **P <* 0.05, ***P <* 0.01, ****P <* 0.001, *****P <* 0.0001, ns means no significant.
